# Genomic analysis of *SBP* gene family in *Saccharum spontaneum* reveals their association with vegetative and reproductive development

**DOI:** 10.1186/s12864-021-08090-3

**Published:** 2021-10-27

**Authors:** Yanhui Liu, Mohammad Aslam, Li-Ang Yao, Man Zhang, Lulu Wang, Huihuang Chen, Youmei Huang, Yuan Qin, Xiaoping Niu

**Affiliations:** 1 Guangxi Key Laboratory of Sugarcane Biology, State Key Laboratory for Conservation and Utilization of Subtropical Agro-Bioresources, College of Agriculture, Guangxi University, Nanning, 530004 China; 2 College of Life Science, Fujian Provincial Key Laboratory of Haixia Applied Plant Systems Biology, Fujian Agriculture and Forestry University, Fuzhou, 350002 China

**Keywords:** Sugarcane, *SBP* genes, Phylogenetic analysis, Expression analysis

## Abstract

**Background:**

SQUAMOSA promoter binding proteins (*SBPs*) genes encode a family of plant-specific transcription factors involved in various growth and development processes, including flower and fruit development, leaf initiation, phase transition, and embryonic development. The *SBP* gene family has been identified and characterized in many species, but no systematic analysis of the *SBP* gene family has been carried out in sugarcane.

**Results:**

In the present study, a total of 50 sequences for 30 *SBP* genes were identified by the genome-wide analysis and designated *SsSBP1* to *SsSBP30* based on their chromosomal distribution. According to the phylogenetic tree, gene structure and motif features, the *SsSBP* genes were classified into eight groups (I to VIII). By synteny analysis, 27 homologous gene pairs existed in *SsSBP* genes, and 37 orthologous gene pairs between sugarcane and sorghum were found. Expression analysis in different tissues, including vegetative and reproductive organs, showed differential expression patterns of *SsSBP* genes, indicating their functional diversity in the various developmental processes. Additionally, 22 *SsSBP* genes were predicted as the potential targets of miR156. The differential expression pattern of miR156 exhibited a negative correlation of transcription levels between miR156 and the *SsSBP* gene in different tissues.

**Conclusions:**

The sugarcane genome possesses 30 *SsSBP* genes, and they shared similar gene structures and motif features in their subfamily. Based on the transcriptional and qRT-PCR analysis, most *SsSBP* genes were found to regulate the leaf initial and female reproductive development. The present study comprehensively and systematically analyzed *SBP* genes in sugarcane and provided a foundation for further studies on the functional characteristics of *SsSBP* genes during different development processes.

**Supplementary Information:**

The online version contains supplementary material available at 10.1186/s12864-021-08090-3.

## Background

Various transcription factors have revealed their critical roles in organism-specific function by activating or suppressing the expression of target genes [1]. The SQUAMOSA promoter binding (like) proteins (SBPs/SPLs) represent a major family of plant-specific transcription factors. SBPs/SPLs proteins share a highly conserved 76 amino acids in length DNA binding domain, also known as SBP binding domain [2]. The first SBP/SPL protein was identified in *Antirrhinum majus*, and this protein could interact with the promoter sequence of the floral meristem gene *SQUAMOSA* [3]. As a multigene family, *SBP/SPL* genes have been characterized from different species ranging from single-cell green algae to multicellular angiosperm [4, 5]. There are 16 *SBP/SPL* genes identified in *Arabidopsis* [6], 19 in rice [7], and 41 in soybean [8]. SBP transcription factors play central roles in various aspects of plant development including [2, 9, 10], flower development [11], leaf development [12], plant hormone signaling transduction [13], vegetative to reproductive phase transition [14, 15]. For example, *AtSPL3* participates in regulating flowering under long photoperiod, and constitutively expressed *SPL3* shows early flowering [6]. *AtSPL8* is a central regulator involved in the regulation of microsporogenesis and megasporogenesis. *spl8* mutant shows pollen sac development defects, and overexpression *SPL8* affects plant fertility by GA-dependent signaling pathway [16]. Moreover, *SPL8* and other *SPL* genes influence gynoecium patterning through mediating auxin homeostasis [17]. In monocot plants, such as rice and maize, *SBP* genes are also reported to modulate essential developmental processes. Overexpression of *OsSPL14* during the reproductive stage significantly promotes panicle branching and increased grain yield [18]. *OsSPL16* is also a regulator of grain size, shape, and quality [19]. *OsSPL3* regulates crown root development [20]. For maize, SBP proteins encoding genes, *unbranched2* and *unbranched3*, affect plant architecture and yield traits by regulating the lateral primordia initiation [21].

Numerous studies have revealed that many development processes mediated by SBP proteins are closely related to miR156. It is reported that miR156 in *Arabidopsis* can complementarily bind to the 3′ UTR of *SPL3* mRNA, and reduce its expression level through translation repression or transcript cleavage [10, 11]. In rice, overexpression of OsmiR156 decreased the expression of *SPL* genes, indicating the conserved interaction relationship between *SPL* and miR156 [22]. Similarly, miR156 targeted *OsSPL16* and *OsSPL13* control grain shape, size and quality in rice [19, 23]. In switchgrass, miR156/SPL4 module controls aerial axillary bud formation and biomass yield [24].

Sugarcane (*Saccharum spontaneum*), one of the most economically valuable plant, is a perennial tropical or subtropical crop, contributing up to about 80% of sugar production and 40% biofuel feedstock in the world [25]. Since 2000 years ago, sugarcane has been cultivated as sugar crop in China and India [26]. This domesticated sugarcane cultivar is a cross between species *S. officinarum* and *S. spontaneum* and accounts for the major genome information to modern sugarcane cultivars [27]. Although the genome information of *S. spontaneum* L. is available [28], little progress has been made in sugarcane germplasm improvement through sexual propagation due to the degeneration of sugarcane reproductive organs [29]. Therefore, unveiling the fundamental mechanism of the sugarcane reproductive developmental process is necessary to develop improved varieties [30].

Concerning recent findings of *SBPs* roles in *Arabidopsis*, rice, and other plants, analysis of *SBP* gene function in sugarcane will undoubtedly accelerate sugarcane germplasm improvement. In this present study, we systematically analyzed the *SBP* gene family of sugarcane for their gene structure, phylogeny, motif and domain composition, miR156 target site, and expression pattern in various tissues and organs. Besides, the interaction between the *SBP* genes and miR156 was critically examined to study their functional relationship during the reproductive stage in sugarcane.

## Results

### Identification and characterization of *SBP* genes in *S. spontaneum*

**Fig. 1:**
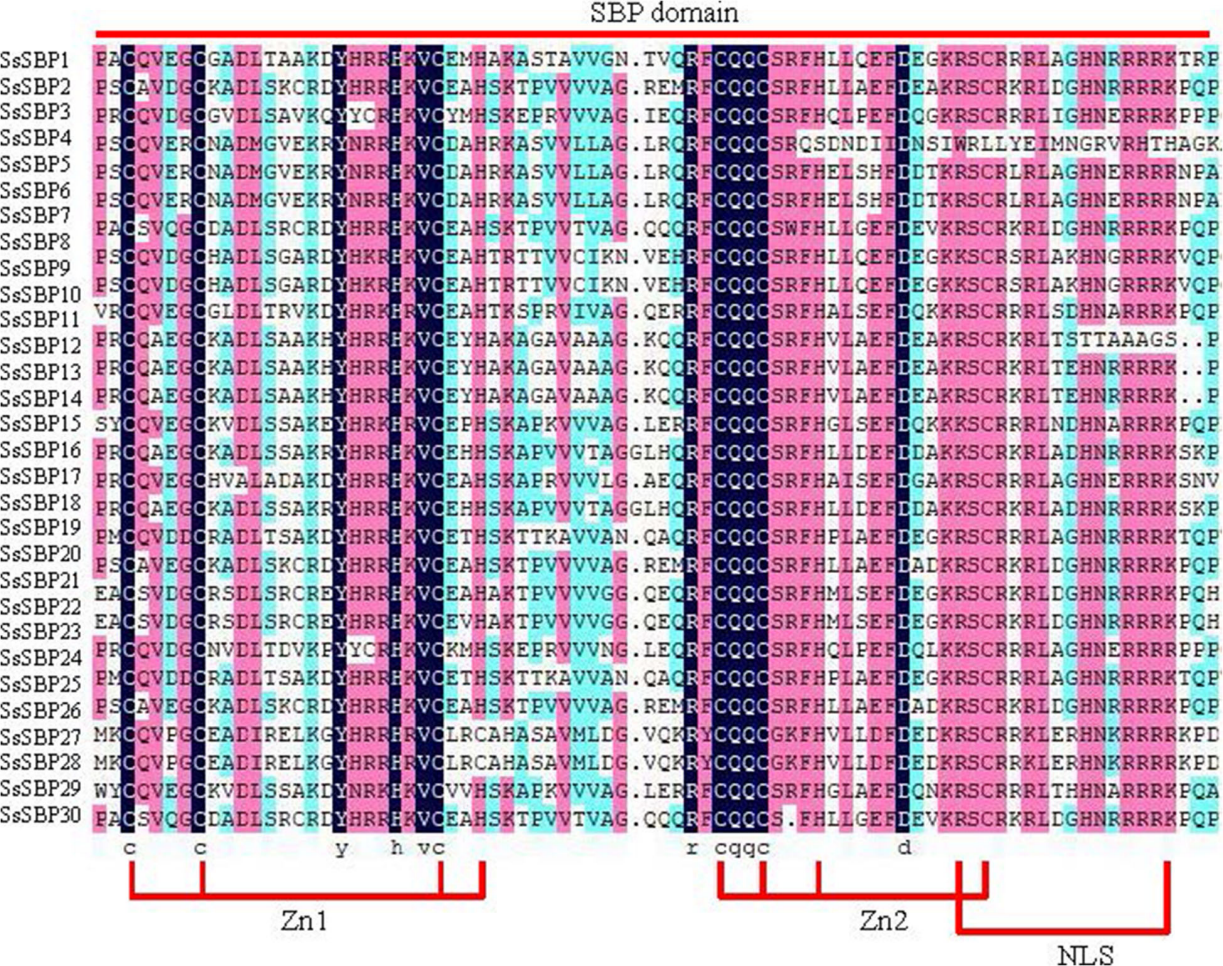
Multiple alignment of the highly conserved SBP domains

To identify of SBP genes in sugarcane, the HMM profile of the SBP domain was used as a query to search the sugarcane genome database and BLASTP program. Initially, 66 putative SBP proteins were identified from the sugarcane genome database. All the resulting sequences were further checked by SMART and pfam tools to confirm SBP domain. Sixteen proteins without SBP (Cys-Cys-His-Cys, Zn2) motif or with incomplete SBP domain were removed. Finally, 50 SBP proteins were identified and used for further analysis. Among them, 13 *SBP* genes had 2, 3 or 4 alleles, including 7 *SsSBPs* with 2 allelic genes, 5 *SsSBPs* with 3 allelic genes and 1 *SsSBP* with 4 allelic genes. We named these *SsSBPs* as *SsSBP1* to *SsSBP30* based on their chromosomal locations and added − 1 to − 4 for their alleles (Table 1). To futher investigate the conserved status of the SBP domain, 30 SBP protein sequences from sugarcane were aligned to predict conserved domains. The alignment results showed that all SsSBP proteins contained the complete SBP domain and possessed the typical characteristics of SBP domain with two Zinc motifs (Zn1 and Zn2) and one nuclear localization signal (NLS) (Fig. 1).

**Table 1:** The characteristics of identified SBP genes in sugarcane

Transcript ID	Name	Chr	Genome locations	ORF	Amino acids	MW (kDa)	pI	GRAVY	Group	Subcellular localization
Sspon.001A0040480	SsSBP1–1	1A	108,429,474–108,435,971	2904	967	105.6349	5.45	−0.303	II	Nuclear
Sspon.001D0044800	SsSBP1–2	1D	109,076,291–109,081,860	2697	898	98.0265	5.53	− 0.313	II	Nuclear
Sspon.002A0013930	SsSBP2–1	2A	29,209,489–29,217,185	1644	547	57.0766	9.25	−0.244	VII	Nuclear
Sspon.002B0011930	SsSBP2–2	2B	28,141,105–28,145,280	1329	442	45.9953	6.53	−0.343	VII	Nuclear
Sspon.002D0010972	SsSBP2–3	2D	23,780,730–23,784,862	1227	408	42.4603	6.53	−0.354	VII	Nuclear
Sspon.002A0015030	SsSBP3–1	2A	31,009,987–31,013,034	1179	392	40.9194	9.14	−0.477	VII	Nuclear
Sspon.002B0012730	SsSBP3–2	2B	29,525,208–29,528,451	1191	396	41.2847	8.98	−0.462	VII	Nuclear
Sspon.002C0016120	SsSBP3–3	2C	35,044,657–35,076,244	3195	1064	116.0996	7.94	−0.351	VII	Nuclear
Sspon.002D0011720	SsSBP3–4	2D	25,045,205–25,048,503	1173	390	40.7902	9.04	−0.491	VII	Nuclear
Sspon.002B0008220	SsSBP4–1	2B	20,690,286–20,695,232	1269	422	42.7577	9.68	−0.315	VI	membrane
Sspon.002C0010600	SsSBP4–2	2C	22,401,265–22,403,347	576	191	19.3214	9.87	−0.399	VI	Nuclear
Sspon.002D0015440	SsSBP4–3	2D	33,849,661–33,853,542	723	240	24.4241	10.32	−0.533	VI	Nuclear
Sspon.002C0010571	SsSBP5	2C	22,345,677–22,347,101	570	189	19.3353	9.96	−0.564	VI	Nuclear
Sspon.002D0015430	SsSBP6	2D	33,845,362–33,846,340	600	199	20.1822	9.96	−0.512	VI	Nuclear
Sspon.003A0000410	SsSBP7–1	3A	1,071,412–1,075,547	1185	394	41.3121	9.15	−0.496	VII	Nuclear
Sspon.003B0003860	SsSBP7–2	3B	7,790,347–7,793,952	1494	497	52.3230	8.94	−0.291	VII	Nuclear
Sspon.003C0005960	SsSBP7–3	3C	11,433,782–11,437,627	1446	481	51.5591	9.57	−0.44	VII	Nuclear
Sspon.003A0019770	SsSBP8	3A	50,439,706–50,445,244	2550	849	94.3843	6.22	−0.423	IV	Nuclear
Sspon.003C0027810	SsSBP9	3C	70,928,512–70,931,044	2604	867	96.6721	7.81	−0.473	IV	Nuclear
Sspon.004A0021810	SsSBP10–1	4A	62,618,391–62,622,079	768	255	27.6747	8.78	−0.693	V	Nuclear
Sspon.004B0022500	SsSBP10–2	4B	66,878,517–66,882,065	1152	383	41.8795	8.67	−0.709	V	Nuclear
Sspon.004A0023470	SsSBP11–1	4A	66,657,938–66,661,498	1458	485	51.7285	9.09	−0.557	V	Nuclear
Sspon.004D0025580	SsSBP11–2	4D	76,179,579–76,181,404	1077	358	38.8064	9.36	−0.673	V	Nuclear
Sspon.004A0023540	SsSBP12	4A	66,840,410–66,841,321	1422	473	50.3841	9.01	−0.551	V	membrane
Sspon.004B0022100	SsSBP13	4B	66,160,113–66,162,260	894	297	31.0155	9.24	−0.16	III	Nuclear
Sspon.004C0023650	SsSBP14	4C	72,419,102–72,423,304	1314	437	45.6945	7.63	−0.296	III	Nuclear
Sspon.004D0023290	SsSBP15–1	4D	70,928,686–70,930,879	936	311	32.8608	9.53	−0.295	III	Nuclear
Sspon.008B0005752	SsSBP15–2	8B	10,803,440–10,806,188	1263	420	44.4747	9.22	−0.588	III	Nuclear
Sspon.008C0006030	SsSBP15–3	8C	13,386,438–13,389,568	1284	427	45.1796	9.23	−0.583	III	Nuclear
Sspon.004D0028460	SsSBP16	4D	82,589,080–82,591,351	1260	419	45.7805	9.52	−0.608	V	Nuclear
Sspon.005A0006871	SsSBP17–1	5A	17,014,877–17,018,085	1185	394	42.8134	7.47	−0.643	III	Nuclear
Sspon.005D0002360	SsSBP17–2	5D	5,016,047–5,019,246	1191	396	42.8995	7.47	−0.641	III	Nuclear
Sspon.005A0007540	SsSBP18–1	5A	18,447,549–18,450,470	1203	400	40.9130	9.50	−0.373	VII	Nuclear
Sspon.005C0003700	SsSBP18–2	5C	9,553,547–9,557,033	1104	367	37.6902	9.48	−0.429	VII	Nuclear
Sspon.005D0002370	SsSBP19	5D	5,036,183–5,040,905	1191	396	43.2850	7.42	−0.716	III	Nuclear
Sspon.006A0002670	SsSBP20–1	6A	6,325,351–6,330,604	2856	951	104.3392	7.10	−0.354	II	Nuclear
Sspon.006D0001140	SsSBP20–2	6D	3,638,437–3,642,110	2298	765	84.0087	8.79	−0.459	II	Nuclear
Sspon.006A0003761	SsSBP21–1	6A	8,766,368–8,771,945	1410	469	48.7223	8.15	−0.456	VII	Nuclear
Sspon.006D0002450	SsSBP21–2	6D	6,732,739–6,738,087	1419	472	49.8476	8.62	−0.479	VII	Nuclear
Sspon.006A0018544	SsSBP22	6A	71,945,300–71,953,194	1065	354	37.6695	9.26	−0.173	VII	Nuclear
Sspon.006A0019261	SsSBP23	6A	75,415,532–75,423,459	1005	334	35.1004	9.93	−0.328	VII	Nuclear
Sspon.006B0001500	SsSBP24–1	6B	3,848,048–3,852,283	1248	415	43.1377	8.93	−0.627	VII	Nuclear
Sspon.006C0001780	SsSBP24–2	6C	4,165,730–4,169,347	1251	416	42.8242	8.87	−0.621	VII	Nuclear
Sspon.006D0000820	SsSBP24–3	6D	2,918,125–2,922,066	1236	411	42.5089	8.87	−0.627	VII	Nuclear
Sspon.006C0002070	SsSBP25	6C	4,855,835–4,856,954	897	298	30.4877	9.80	−0.63	II	Nuclear
Sspon.006D0002410	SsSBP26	6D	6,577,854–6,582,459	726	241	26.0042	8.71	−0.741	VII	Nuclear
Sspon.007A0010700	SsSBP27	7A	22,289,512–22,304,260	1998	665	72.2692	5.37	−0.438	I	Nuclear
Sspon.007A0010740	SsSBP28	7A	22,398,323–22,403,566	1311	436	47.2836	5.56	−0.463	I	Nuclear
Sspon.008B0001960	SsSBP29	8B	2,868,989–2,873,212	1272	423	45.4750	9.32	−0.382	V	Nuclear
Sspon.ctg0104090	SsSBP30	tig00011976	14–4187	1065	354	38.0538	9.30	−0.512	VII	Nuclear

The detailed information about the SsSBPs was deduced by ExPASy server, including protein length, molecular weight (MW), theoretical isoelectric point (pI) and the grand average of hydropathicity (GRAVY). The length of the *SsSBPs* ORF region varied from 570 bp (*SsSBP5*) to 3195 bp (*SsSBP3–3*) and the protein lengths ranged from 189 to 1064 amino acids. The MW of the proteins ranged from 19.3214 to 116.09958 kDa. The pI ranged from 5.37 to 10.32, and the values of GRAVY were all negative, suggesting that all SsSBPs are hydrophilic. Moreover, the subcellular localization of 50 SsSBP proteins was predicted by ProtComp software and found that all SsSBP proteins localized in the nucleus except SsSBP4 and SsSBP12 proteins, which have no NLS signal, and localize in the cell membrane (Fig. S1; Table 1).

### Phylogenetic analysis of the *SBP* gene families

**Fig. 2:**
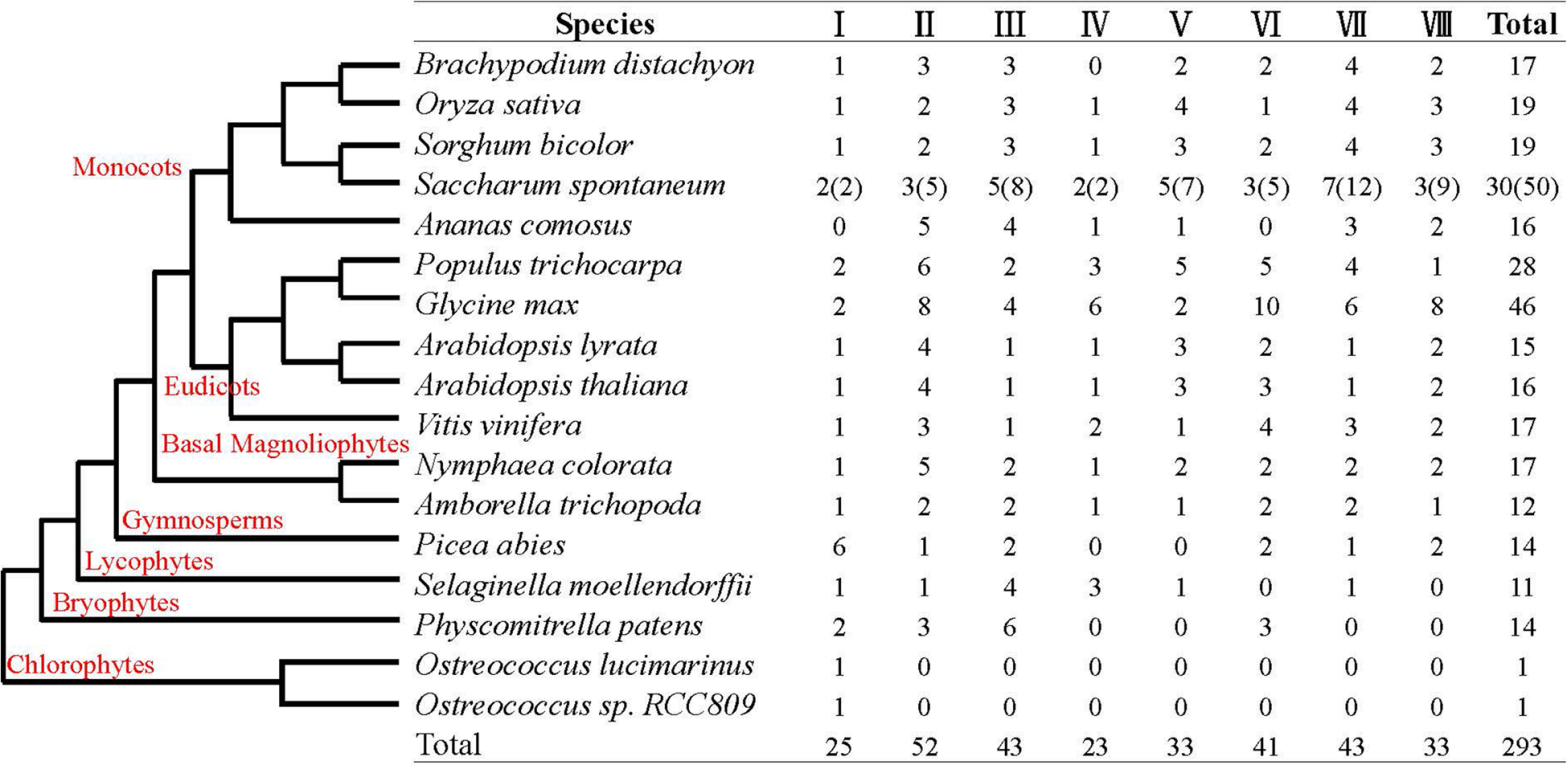
Distribution of *SBP* genes in 17 species

**Fig. 3:**
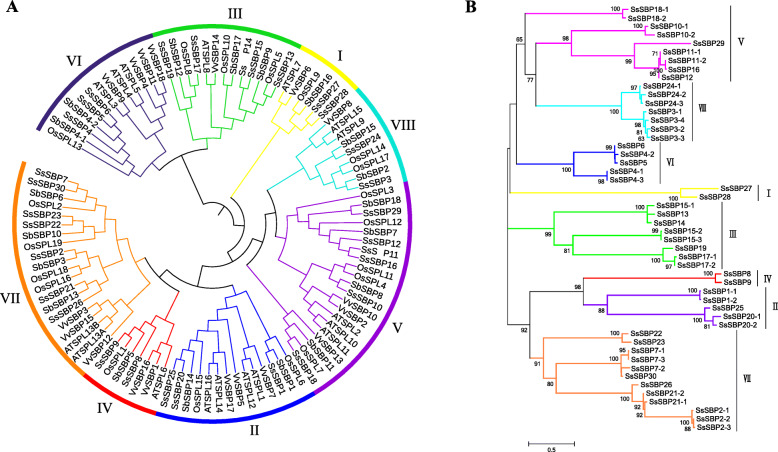
Maximum likelihood phylogenetic tree of *SBP* gene family

We selected a total of 293 *SBP* homologs from 17 representative species from 7 green plant families, including chlorophytes, bryophytes, lycophytes, gymnosperms, basal magnoliophytes, eudicots and monocots, for phylogenetic analysis of SBP. Among them, both *Ostreococcus sp. RCC809* and *Ostreococcus Lucimarinus* in green algae had only one *SBP* gene; 46 *SBP* genes exist in soybean. In comparison to the number of genes in these species, *SBP* genes in *S. spontaneum* showed an obvious expansion in the number of genes (Fig. 2). To gain further insight into the phylogenetic relationship of *SsSBP* genes, a phylogenetic tree was constructed using SBP proteins from *Arabidopsis, Vitis vinifera*, *Ananas comosus*, *Sorghum bicolor* and *Oryza sativa* (Fig. 3). *SBP* genes from these different species could be classified into 8 groups (I to VIII), and SBP proteins also tend to cluster the similar group. As expected, SsSBPs exhibited a closer relationship with the SBP proteins from *S. bicolor* and *O. sativa*. Group V and VII contained maximum *SsSBP* genes, where *SBP* genes from *S. bicolor* and *O. sativa* were also grouped. While the group I contained only 2 members of *SsSBP* genes formed the smallest group. This result was in agreement with the conservation analysis of the SBP proteins in other plants like *Arabidopsis*, grape, rice and sorghum. For example, a relatively high homologous genes, *AtSPL6* / *SbSBP5* / *OsSPL1* / *SsSBP8* / *SsSBP9* clustered in one evolutionary branch (Fig. 3A). In addition, a ML phylogenetic tree was also constructed based on gene sequence similarity of 50 SsSBP proteins. The result indicated that the alleles of each *SsSBP* gene cluster in the same group, indicating that their sequences have high homology (Fig. 3B).

### Structure characterization of *SBP* genes in *S. spontaneum*

**Fig. 4:**
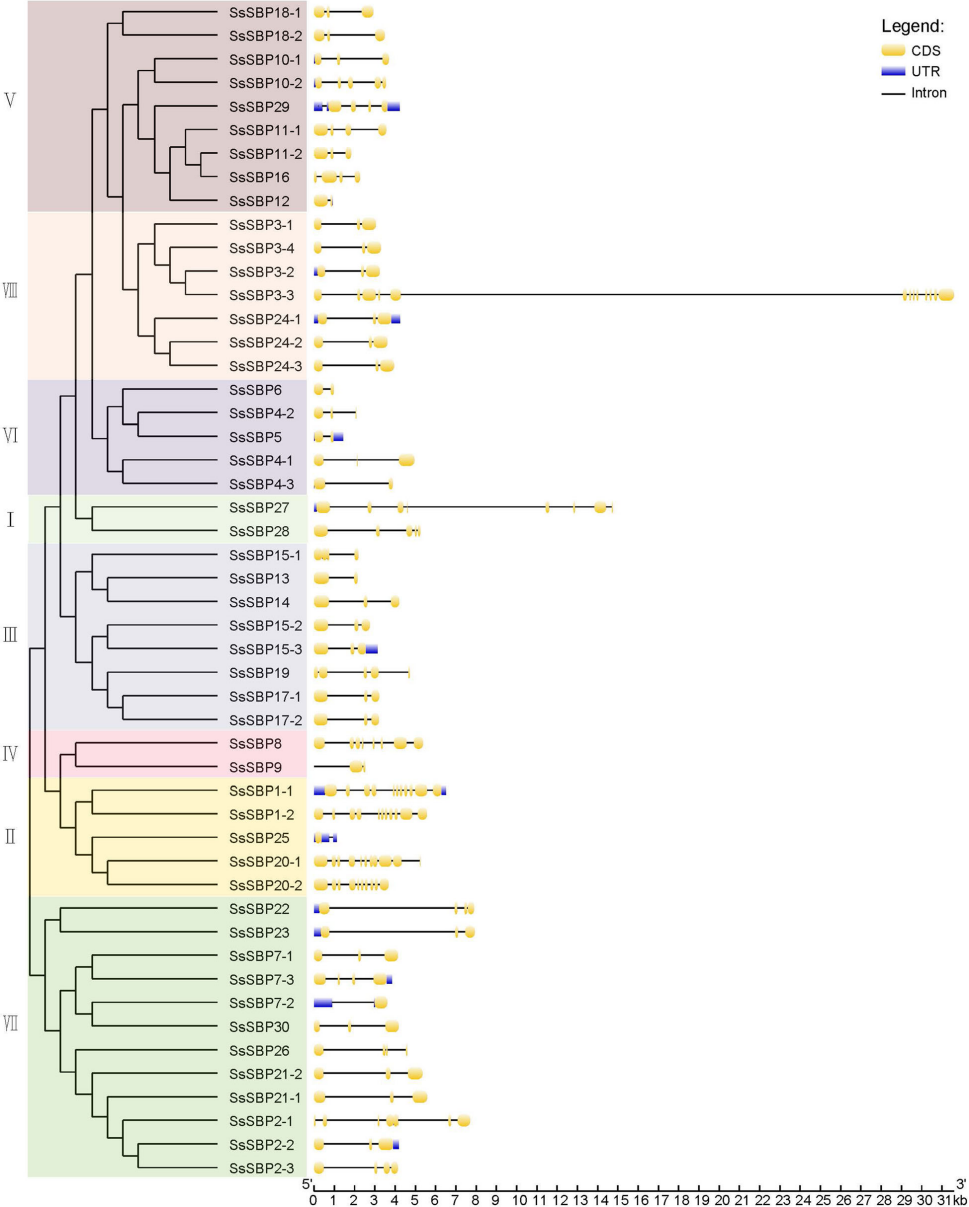
The exon/intron structure of sugarcane *SBP* genes

To better understand the genetic diversity of the *SsSBP* genes, the coding sequence of each gene was compared with their corresponding genomic sequence. The result revealed that the exon of *SsSBP* genes ranged from 2 to 13 in number (Fig. 4). *SsSBP* genes in group I contain 4–7 introns, group V contains 2–4 introns, group VI contains 1–2 introns, most of *SsSBP* members in group VIII contain 2 introns. Interestingly, alleles of *SsSBP* genes (*SsSBP1–1/2*, *SsSBP3–1/2/4*, *SsSBP4–1/2*, *SsSBP15–2/3*, *SsSBP17–1/2*, *SsSBP18–1/2*, *SsSBP20–1/2*, *SsSBP21–1/2* and *SsSBP24–1/2/3*) had the same number of exon/introns, although the length of introns varied. Some alleles possessed different exon/intron numbers (*SsSBP2–1/2/3*, *SsSBP7–1/2/3*, *SsSBP10–1/2*, *SsSBP11–1/2*). The *SsSBP3* allele gene *SsSBP3–3* possessed the largest number (13) of exons, but the *SsSBP3* alleles *SsSBP3–1/2/4* only had 3 exons (Fig. 4). These results indicate that the number of exon and intron are diverse in different groups yet nearly consistent within the same group.

**Fig. 5:**
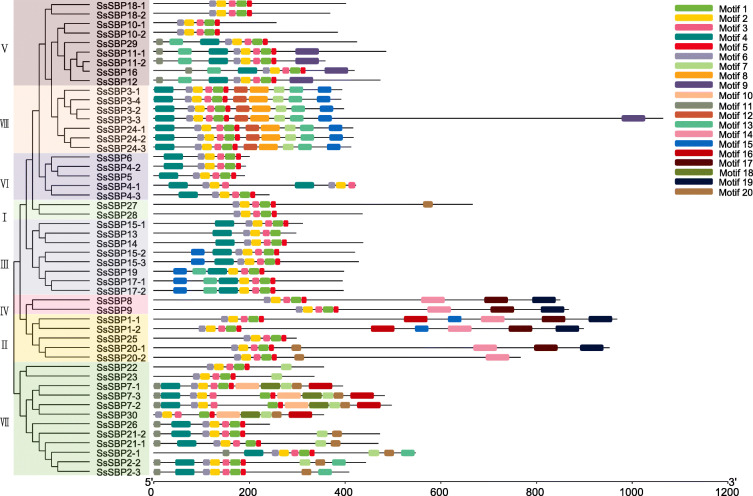
Motif composition of sugarcane SBP proteins. The motif numbers from 1 to 20 are displayed in different colored boxes

The *SsSBP* genes clustered into the same group exhibited similar structure and possessed a similar motif sequence. A total of 20 motifs were identified in SsSBP proteins, designated as motif 1–20 (Fig. 5, Fig. S2). The result expectedly showed that all SBP members contain Motif 1, Motif 2, Motif 3, Motif 5 and Motif 6, which was annotated as the SBP domain. Most of SBP members within the same group exhibited similar motif composition and exon-intron structure. In contrast, some motifs were found to be specific to one or two groups of SsSBP proteins. Motif 8 only appeared in group VIII. Motif 14, Motif 17 and Motif 19 were only present in group II, suggesting that proteins in these groups may have a specific biological function under a given condition. At the same time, the divergence among the different groups indicated their diverse functions.

### Chromosome distribution and gene duplication of *SsSBP* genes

**Fig. 6:**
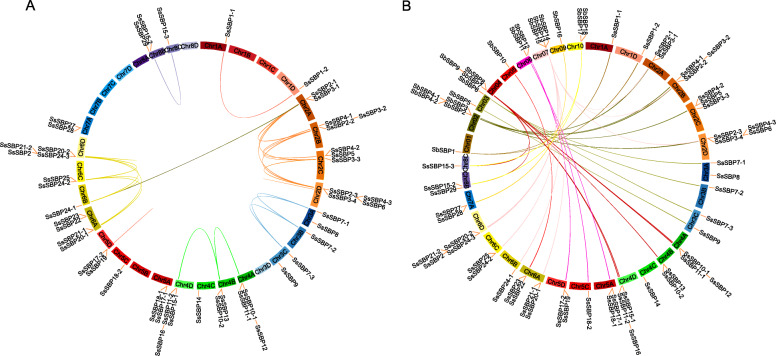
Gene location and synteny in sugarcane. **A** Synteny analysis of *SBP* genes within sugarcane. **B** Synteny analysis of *SBP* genes between sugarcane and sorghum

The chromosome distribution information of *SsSBP* genes revealed that 49 of the 50 *SsSBP* genes are located to the eight chromosomes of *S. spontaneum*, with the *SsSBP30* mapped to the unanchored scaffolds (Fig. 6). On chromosomes 1 and 7, only two *SsSBP* genes were found. Chromosomes 3 and 5 contain five *SsSBP* genes. Chromosome 2 had the maximum number of *SsSBP* genes with 12 members, followed by chromosome 6 with 11 *SsSBP* genes. In addition, 27 synteny gene pairs were identified in sugarcane using MCScanX software, with 24 pairs of alleles and 3 pairs of nonalleles. It should be defined as a tandem duplication event if a chromosomal region within 200 kb containing two or more genes [31]. According to this criterion, only two tandem duplications (*SsSBP4–3*/*SsSBP6* and *SsSBP17–2*/*SsSBP19*) were noticed (Fig. 6A, Table S3). These results indicate that segmental duplication events might significantly contribute to the *SsSBP* gene expansions than tandem duplication.

To further analyze the evolutionary process of *SsSBP* genes, a comparative analysis of genome synteny blocks between *S. spontaneum* and *Sorghum bicolor* was conducted. Sorghum is the closest related diploid to sugarcane, and the comparison of gene structures between these two species provided clues to the evolutionary gene events caused by polyploidization. A total of 37 syntenic gene pairs between *S. spontaneum* and *S. bicolor* were found (Fig. 6B, Table S3). To further understand the evolutionary forces on *SsSBP* genes, the ratio of the synonymous (*Ks*) and nonsynonymous (*Ka*) substitutions rate (*Ka*/*Ks*) was calculated for estimating the selection pressure of homologous genes, where *Ka*/*Ks* < 1 indicates purifying selection, *Ka*/*Ks* = 1 means neutral selection and *Ka*/*Ks* > 1 indicates positive selection [32]. In this study, with the exception of three gene pairs *SsSBP4–1*/*SsSBP5* (1.0379), *SsSBP3–2*/*SsSBP3–4* (1.98497), *SsSBP24–2*/*SsSBP24–3* (1.00893), *Ka*/*Ks* ratios of *SsSBP* homologous genes were less than 1, indicating that these genes probably underwent a purifying selection (Table S2). Similarly, most *Ka*/*Ks* values of sorghum genes were also less than 1, suggested that *SBP* genes of these two close species underwent a strong purifying selection to reduce adverse mutations after duplication during the evolutionary process (Table S3).

### miR156 target prediction, distribution and expression pattern analysis

**Fig. 7:**
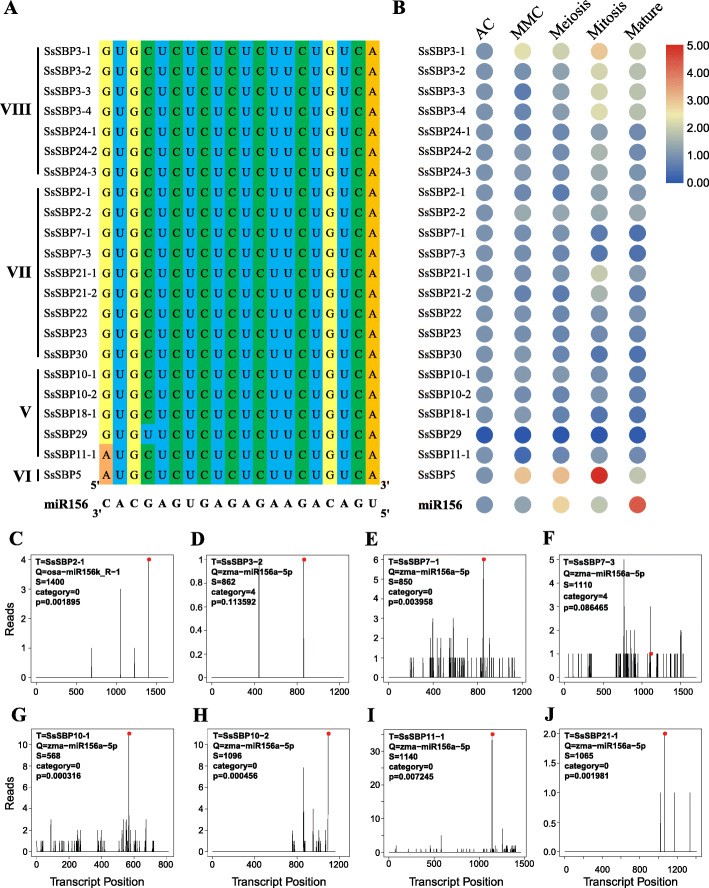
Sequence alignment and expression patterns of miR156 compare with *SsSBP* genes. **A** Sequence alignment of the miR156 complementary sequences with the target sites in *SsSBP* genes. **B** The expression patterns of miR156 and their targets in different tissue samples. **C**-**J** The confirmation of miR156s and their targets by degradome ananlysis

Previous studies showed that miR156 complementarily binds to *SBP* genes either at the coding or 3’UTR region and reducs gene expression level through translation repression or transcript cleavage [11, 24]. In this present study, 22 *SsSBP* genes were found as the targets of miR156, and these genes were mainly distributed in groups V, VI, VII and VIII (Fig. 7A). Among these *SsSBP* genes, miR156 complementary sequences were at their coding regions except *SsSBP5* where miR156 binding in the 3′-UTR (Fig. 7A; Fig. S3). Interestingly, the allelic genes *SsSBP3–1*, *SsSBP3–2*, *SsSBP3–3* and *SsSBP3–4* were all the targets of miR156 (Fig. 7A). Although there are 20 miRNA members of *Saccharum sp*., only one putative miRNA156 in *S. spontaneum* is represented in the miRbase database (https://www.mirbase.org/ v 22.1). Therefore, we performed a genome-wide study and found 29 members of miR156 genes in *S. spontaneum* genome. These *Ssp-miR156* genes were mainly distributed on chromosomes 2, 3, 4, 5, 6, 8, except for chromosomes 1 and 7 (Fig. S4; Table S4). *Ssp-miR156a*, *Ssp-miR156f*, *Ssp-miR156j* and *Ssp-miR156k* as alleles were localized on chromosome 2A. Chromosome 3A possesses 4 *Ssp-miR156a* alleles. Chromosome 3B contains 3 miR156 members (2 *Ssp-miR156a* and 1 *Ssp-miR156l*). Chromosome 4A has 4 miR156 members (3 *Ssp-miR156a* and 1 *Ssp-miR156i*), followed by 2 and 3 members on chromosome 4B (*Ssp-miR156a* and *Ssp-miR156d*) and 5A (2 *Ssp-miR156a* and 1 *Ssp-miR156e*), respectively. Two *Ssp-miR156* were on chromosome 8A (*Ssp-miR156e* and *Ssp-miR156k*) and 8D (2 *Ssp-miR156b*). Only 1 *Ssp-miR156* was found on chromosome 2C (*Ssp-miR156b*), 2D (*Ssp-miR156b*), 3C (*Ssp-miR156a*), 6A (*Ssp-miR156a*) and 6B (*Ssp-miR156a*). However, no *Ssp-miR156* members was found on chromosomes 1 and 7 (Fig. S4; Table S4).

To further gain insight into the role of miR156 during female gametophyte development, we studied the miR156-SBP module during female gametophyte development. The results showed that the expression level of miR156 was mostly enriched in the mature stage of female reproductive development, and relatively low expression levels were found during the stages of AC (Archesporial Cell) to MMC (Megaspore Mother Cell). Generally, the expression level of miR156 increased from the initial stage to the mature stage of the female gametophyte by sRNA-seq analysis (Fig. 7B; Table S5). In addition, the expression profiles of *Ssp-miR156* precursors were also quantitatively verified using RT-PCR and qRT-PCR analysis. The results for the relative expression of *Ssp-miR156* were consistent with the sRNA-seq data (Fig. S5). On the contrary, the expression level of target *SsSBP* genes was mostly decreased during the female gametophyte development stages, such as the target *SsSBP11–1*, *SsSBP21–2*, *SsSBP22* and *SsSBP30* (Fig. 7B).

To verify the authenticity of miR156-SBP module in sugarcane, we performed the degradome analysis and found that miR156 family members target the *SsSBPs.* The miR156k could bind the site 1400 bp of its target *SsSBP2–1* (Fig. 7C). Similarily, miR156a binds on the *SsSBP3–2* (site 862) (Fig. 7D), *SsSBP7–1* (site 850) (Fig. 7E), *SsSBP7–3* (site 1110) (Fig. 7F), *SsSBP10–1* (site 568) (Fig. 7G), *SsSBP10–2* (site 1096) (Fig. 7H), *SsSBP11–1* (site 1140) (Fig. 7I) and *SsSBP21–1* (site 1065) (Fig. 7J). Taken together, these results suggest that miR156-*SBP* module is highly conserved, and the regulation pattern has diverged in different species.

### Expression profiles analysis of *SsSBP* genes

**Fig. 8:**
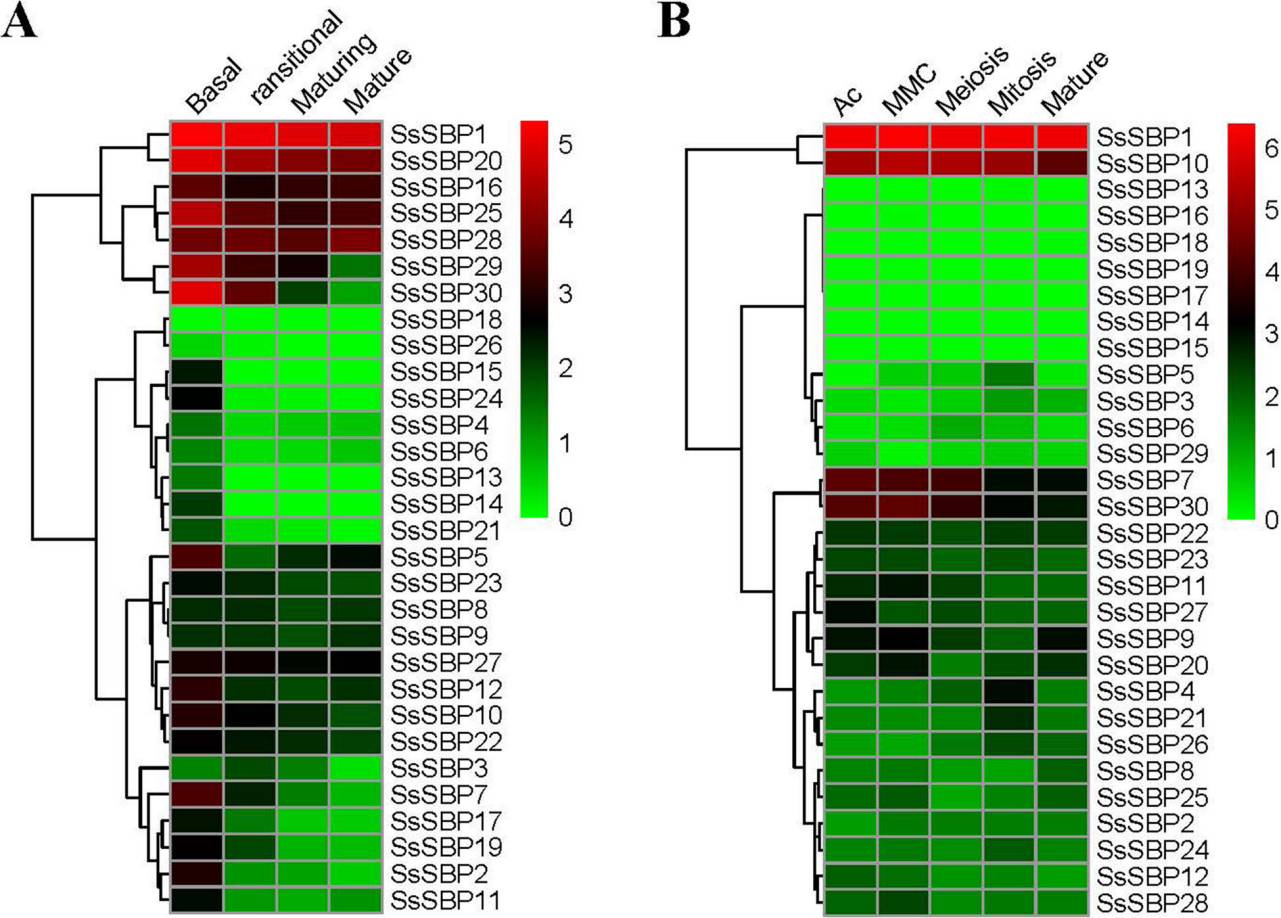
Expression profiling of *SsSBP* genes in different sugarcane tissues. The color bar shows the gene expression with log2 (FPKM+ 1). Green colors indicate low expression and red colors indicate high expression

To study spatiotemporal expression patterns of *SsSBP* genes, RNA-seq data of different organs and tissues were analyzed. The expression level of *SsSBP* genes of leaf development and female reproductive organs is shown by heatmap representation (Fig. 8). As illustrated in Fig. 8A, *SsSBP4*, *SsSBP6*, *SsSBP13*, *SsSBP14*, *SsSBP18*, *SsSBP21* and *SsSBP26* sustained low expression level in sugarcane leaf gradient segments, while *SsSBP1* and *SsSBP20* showed high expression in the leaf gradient segments. The transcript levels of *SsSBP7*, *SsSBP10*, *SsSBP19*, *SsSBP22*, *SsSBP28* and *SsBP29* decreased gradually from base to mature zone of leaf in sugarcane, showed that gene expression decreased following the maturing leaf (Fig. 8A, Table S6).

To investigate the *SsSBP* genes involvement in sugarcane female reproductive organ development, the transcription level of all *SsSBP* genes was extracted from RNA-seq data of sugarcane female reproductive organs. The heat map represents expression levels in the lines at five developmental stages shown in Fig. 8B. Many *SsSBP* genes showed different expression patterns among these five development stages. *SsSBP1* and *SsSBP10* were highly expressed in different stages of female gametophyte development. The transcripts of 7 *SsSBP* genes (*SsSBP13*, *SsSBP14*, *SsSBP15*, *SsSBP16*, *SsSBP17*, *SsSBP18*, *SsSBP19*) were zero in all these samples. The expression level of *SsSBP7* and *SsSBP30* showed differential expression during the female gametophyte development, revealing that these two genes may play an important role in AC and MMC stages (Fig. 8B, Table S7).

We also performed qRT-PCR experiments to confirm the expression level of some *SsSBP* genes in those different female developmental stages. As shown in Fig. S6, the results of qRT-PCR data were highly consistent with the RNA-seq data for the relative expression of SsSBP genes during the female gametophyte development. Further studies may focus on the role of these genes on female reproductive development.

## Discussion

Sugarcane (*S. spontaneum*) has been widely domesticated and cultivated for thousands of years for its excellent economic values. It has become essential industrial material for sugar sources [25, 26]. The high quantity of genome data and abundance of increasing high-throughput transcriptome data make it possible to explore gene functions in non-model plants like *Saccharum spp*. Although the genome information of *S. spontaneum* L. is available, little progress has been made in sugarcane germplasm innovation and improvement due to the degeneration of sugarcane reproductive organs [28, 29]. Previous studies revealed that *SBP* genes play crucial roles in plant development, especially in flower development, signaling transduction, and vegetative to reproductive phase transition [13–15]. However, the functions of *S. spontaneum SBP* genes remain unknown, although 17 SPLs were identified in sugarcane without taking alleles into account [33]. As for sugarcane genomic autopolyploidization, we conducted the genome-wide identification of *SBP* genes and their alleles in *S. spontaneum*, which resulted in the identification of 30 *SBP* genes (Fig. 1, Table 1). The number of *SBP* genes in *S. spontaneum* was similar to that in *P. trichocarpa* (28), *O. zativa* (19), and *S. bicolor* (19), but smaller than that in *G. max* (46), indicating that *SBP* genes in different species underwent different gene duplication events. Based on phylogenetic and gene structure analysis, *SsSBP* genes could be divided into eight groups (group I-VIII), which is consistent with the results of previous studies on *SBP* genes [34].

In general, the members of *SBP* genes clustered into a subgroup shared similar gene structure and functions, suggesting these genes underwent common evolutionary origins. In other words, gene duplication events (segmental and tandem duplication) are the major driving forces for evolution and gene expansion by which many paralogous gene pairs are produced and could help organisms cope with different developmental processes [35]. In our study, a total of 27 duplication events were found in *SsSBP* genes, consisted of segmental duplications and tandem duplication (Fig. 6). The *Ka/Ks* ratio is reported as the criterion for estimation the gene duplication. The *Ka/Ks* ratio of a given > 1 means that the gene has experienced positive selection, = 1 suggests neutral selection and < 1 indicates purifying selection. Based on the values of *Ka/Ks* ratio, all the *SsSBP* gene pairs were duplicated under purifying selection except gene pairs *SsSBP4–1*/*SsSBP5* (1.0379), *SsSBP3–2*/*SsSBP3–4* (1.98497), *SsSBP24–2*/*SsSBP24–3* (1.00893) (Table S3). The diversity of *SsSBP* genes is likely to be motivated by gene duplication and genomic structure variation during the evolutionary process.

Up to now, there is little functional information on the SBP genes of sugarcane. Generally, the gene functions, to a large extent, are correlated to their expression patterns. In this present study, the expression levels of 30 *SsSBP* genes were examined across the four different leaf gradient segments and five female gametophyte development stages (Fig. 7). Most *SsSBPs* were predominantly expressed in the initial developmental stages of either leaf development or female gametophyte development. These results were similar with other species in the apical meristem, including apical buds inflorescences and flower buds [9, 10, 22]. Among the *SBP* genes in *Arabidopsis*, *AtSPL1* and *AtSPL12* expressed highly in inflorescences and overexpression of these two genes enhanced the inflorescence thermotolerance [36]. *AtSPL2*, *AtSPL9*, *AtSPL10*, *AtSPL11*, *AtSPL13* and *AtSPL15* were reported to control the determination of leaf shape and the transformation of vegetative to reproductive stages [37]. Interestingly, the evolutionary analysis showed that *AtSPL1* and *AtSPL12* are highly orthologous to *SsSBP* genes in group II, including *SsSBP1*, *SsSBP20* and *SsSBP25*. *AtSPL2*, *AtSPL9* and *AtSPL10* are orthologous to *SsSBP* genes in the group V with *SsSBP10*, *SsSBP11*, *SsSBP12*, *SsSBP16*, and *SsSBP29*. Based on the belief that homologous genes perform similar functions. *SsSBP1* and *SsSBP10*, which were expressed highly in female gametophyte stages, would be involved in the development of female reproductive organs in sugarcane. Three genes *SsSBP1*, *SsSBP20*, *SsSBP25* grouped with *SsSBP16*, *SsSBP29* and *SsSBP30*, which are orthologous to *AtSPL2*, *AtSPL9* and *AtSPL10*, expressed highly in the sugarcane leaves, confirming their roles in the regulation of leaf development. Certainly, additional studies need to be performed to confirm the potential roles in female gametophyte development (for *SsSBP1* and *SsSBP10*) and leaf development (for *SsSBP1*, *SsSBP20*, *SsSBP25*, *SsSBP16*, *SsSBP29* and *SsSBP30*).

In addition, miR156/SBP module has been reported to govern many aspects of plant growth and development [10, 17, 24, 38]. Overexpression of miR156 in *Arabidopsis* significantly repressed the *SPL* transcription and resulted in the loss of apical dominance, leading to dwarfism, an increase in total leaf number, and plant biomass [39]. Meanwhile, the expression levels of the target *SBP* genes of miR156 were suppressed in the miR156 overexpressing plants [10, 37]. In the present study, the transcript level of miR156 was abundant in the mature stage of female reproductive development (Fig. 7B). In contrast, most putative target *SsSBP* genes predicted miR156 target sites showed lower expression levels in these tissues (Fig. 7B). These results suggested that the transcript of miR156 is negatively correlated with the expression of most *SsSBP* genes (Fig. 8). All together, our results revealed that miR156/SBP module could be used as an important tool to genetically improve crop architecture and productivity.

## Conclusion

**Fig. 9:**
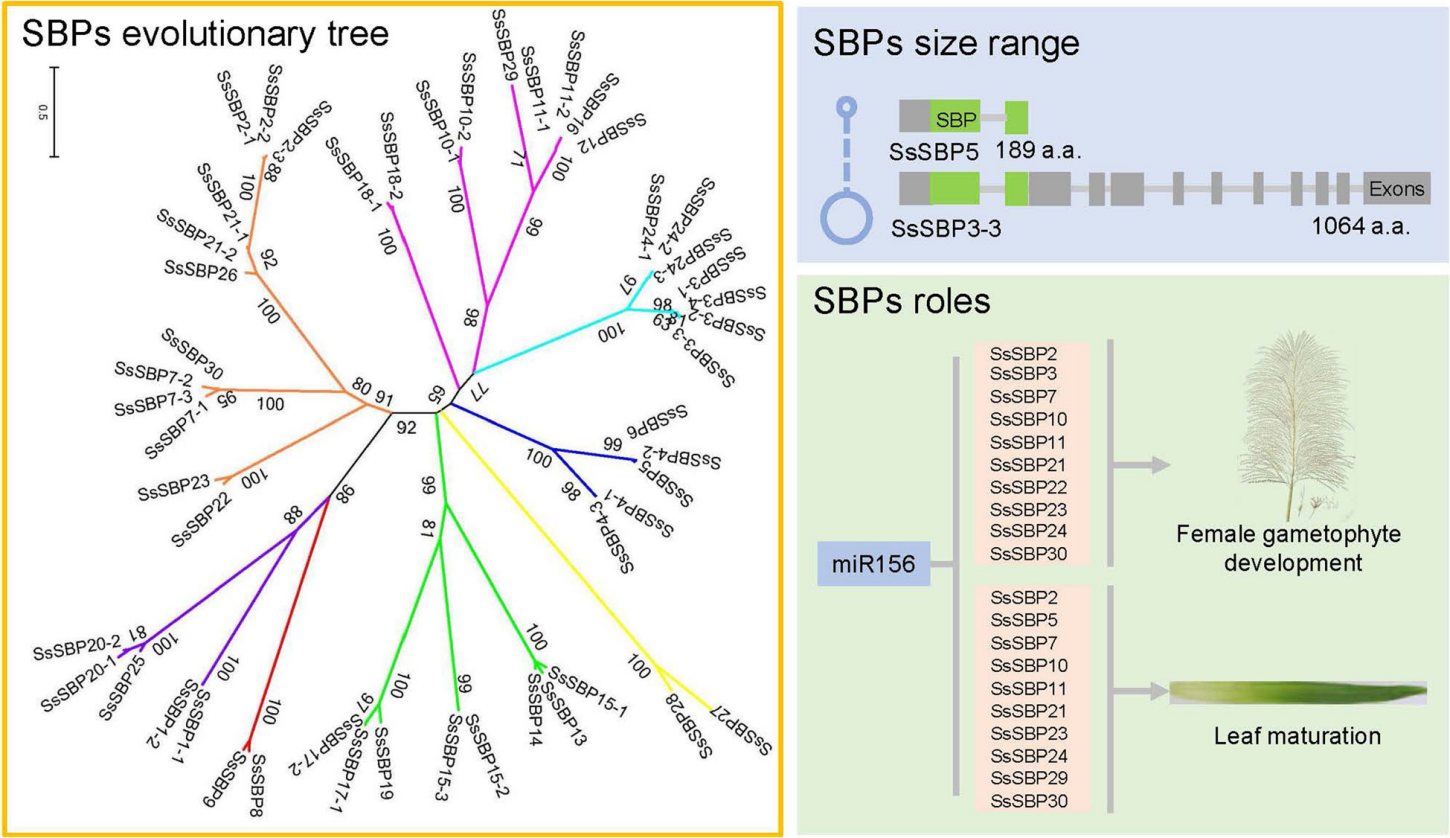
A summary work model of this study for SBP genes in sugarcane

A total of 30 *SBP* genes were identified in sugarcane (*S. spontaneum*) by genome-wide analysis. These *SsSBP* genes were comprehensively characterized and classified into eight groups. The phylogenetic analysis showed that these genes shared orthologous relationships of SBP members from Arabidopsis and rice. The spatiotemporal expression patterns of these *SsSBP* genes in different tissues indicate that *SsSBP* genes may regulate the leaf and female gametophyte development. Our results also showed that miR156 targeted many SsSBP genes. The expression level of miR156 was enriched in the female reproductive mature stages. The different expression levels between the miR156 and *SsSBP* genes in diverse tissues suggested that miR156/SBP module plays a crucial role in the leaf and female gametophyte development processes (Fig. 9). Taken together, our study provides the foundation for future in-depth elaboration of the potential functions of the *SBP* genes in the growth and development of sugarcane.

## Methods

### Identification and annotation of *SBP* genes in sugarcane

Sugarcane genome data, CDS, protein sequence and annotation data were downloaded from the sugarcane Genome database (http://sugarcane.zhangjisenlab.cn/sgd/html/index.html) [28]. *Arabidopsis* and other species sequences were searched and downloaded from Phytozome v13 (https://phytozome.jgi.doe.gov/pz/portal.html) [40]. To identify the *SBP* genes in sugarcane, the HMM profile of the SBP domain (PF03110) was downloaded from the Pfam database (http://pfam.xfam.org/) [41] and used as the query to search the sugarcane genome database. *SBP* homologs were obtained by running a local BLASTP search using the *Arabidopsis* and rice *SBP* sequence as a query against the given protein database with an E-value cutoff of 10^− 5^. The candidate genes were further confirmed by SMART server (http://smart.embl-heidelberg.de/). Sequences without the complete SBP domain were deleted. Finally, all the candidates were confirmed by multiple sequence alignments using DNAMAN software to ensure they contained the SBP domain. ExPASy (https://www.expasy.org/) [42] server was used to calculate the detailed information about the SsSBPs in sugarcane, such as molecular weights (MW), isoionic point (pI), and the grand average of hydropathicity (GRAVY). The subcellular localization of the SBP proteins identified was obtained using the ProtComp (v.9.0) software (http://www.softberry.com/).

### Gene structure, sequence alignments and phylogenetic analysis of *SsSBP* genes

The exon/intron structure of *SBP* genes was analyzed using Gene Structure Display Server (http://gsds.cbi.pku.edu.cn/index.php) [43] by comparing their coding and genomic sequences. Using BLASTP program to search homologous gene pairs among sugarcane and sorghum with the parameter of e-value = 1e-10. The estimation of selection and substitution rates, the non-synonymous (Ka), synonymous (Ks) and Ka/Ks substitution ratios of the homologous gene pairs of sugarcane and sorghum were calculated by the easy Ka/Ks calculation program. MCScanX software [44] was used to detect the gene synteny and collinearity in sugarcane, and the *SBPs* locations were shown using Circos software [45]. Multiple sequence alignment of SBP protein sequence from *Arabidopsis*, rice, and sorghum was conducted using the MUSCLE in MEGA (v.6.0) [46]. A phylogenetic tree was constructed using RAxML software (http://www.phylo.org/index.php/) using the maximum likelihood (ML) method with bootstrap 1000 replications. The phylogenetic tree was displayed and manipulated using the Interactive tree of life (iTOL, https://itol.embl.de/) [47–49].

### Conserved motif identification, miR156 target site prediction and distribution

The conserved motifs of SsSBP proteins were identified using the online program MEME (http://meme-suite.org/tools/meme) [50] with the default setting parameters: maximum number of motifs to find was 20; minimum width of motif was 6 and maximum width of motif was 50. The sequence logos of the SsSBP domain were showed by TBtools [51]. To predict the putative target sites of miR156, the cDNA sequences of *SsSBP* genes were analyzed using psRNATarget tool (http://plantgrn.noble.org/psRNATarget/). The chromosome location information of the *Ssp-miR156s* and *SBPs* were searched in sugarcane genome databases, and MapInspect software was used to generate chromosomal distribution information.

### Plant material and sample preparation

The sugarcane (*S. spontaneum* L.) cultivar Yuetang 91–976 was grown and collected by State Key Laboratory for Conservation and Utilization of Subtropical Agro-Bioresources (Guangxi, China), and all samples from this cultivar was adopted for all experiment. When the plants reached the age of florescence stage, five different stages of the sugarcane female gametophyte development (i.e., AC, MMC, Meiosis, Mitosis and Mature) were collected. All samples were harvested as three biological replicates, which were quick-freeze with liquid nitrogen and stored at ultra-low temperature to facilitate the extraction of RNA.

### RNA extraction, expression profiles and qRT-PCR analysis

Total RNA was isolated by the Omega Total RNA kit II (R6934–02, USA). The evaluation of RNA quality was performed by the gel electrophoresis and 2000 spectrophotometer assessment at 260 nm (NanoDrop, Thermo Fisher Scientific), and Illumina sequencing was done using the method of Zhao et al. (2018).

For qRT-PCR analysis, the cDNA was synthesized using the ThermoScript RT-PCR kit (Life Technologies) in a 20 μL volume reaction under the program: 42 °C for 15 min and 85 °C for 15 s. According to the SYBR Premix RT reagent kit system (TaKaRa, Dalian, China), the reaction contains 1 μg RNA prior to qRT-PCR.

To understand the expression profiles of *SBP* genes, the RNA-seq data of leaf development were downloaded from the Saccharum Genome database (http://sugarcane.zhangjisenlab.cn/sgd/html/index.html). The RNA-seq data of female reproductive development have been deposited in the European Nucleotide Archive (ENA, accession number PRJEB44944). Different leaf developmental stages, including basal zone, a transitional zone, a maturing zone, a mature zone [52, 53], and the female reproductive development stages, AC, MMC, Meiosis, Mitosis and Mature, were used for the study. The RNA-seq raw reads were filtered by Trimmomatic software with default parameters to obtain clean reads. The clean reads were mapped to the reference genome using Hisat2 [54]. Gene expression was calculated by Cufflinks software [55]. The log2-transformed RPKM value of the expression patterns of *SsSBP* genes was used to generate the heatmap using the pheatmap package in R software. The expression pattern of miR156 was calculated by count values according to the miRNA-seq data.

To further confirm the expression profiles of the *SsSBP* genes, qRT-PCR assays were performed in different female reproductive development stages. qRT-PCR was conducted in CFX96 Real-Time System (Bio-Rad) using SYBR Green (TaKaRa) according to the instructions. Each reaction contains 12.5 μL SYBR mixture, 1.0 μL specific primer and 1 μg sample template. Three replicate reactions were performed for each sample under the following program: 95 °C for 30s; 40 cycles of 95 °C for 5 s; 60 °C for 30 s. The primers used in this study are listed in Table S1.

## Supplementary Information

**Additional file 1.**

## Data Availability

All data generated or analyzed during this study are included in this published article and its supplementary information files. The sequencing data of miRNA that support the findings of this study have been deposited in the NCBI SRA database with BioProject accession no. PRJNA723681 (https://dataview.ncbi.nlm.nih.gov/object/PRJNA723681?reviewer=vd8eph3l6oqf79jo3ta52181gr), and the RNA-seq data of female reproductive development have been deposited in the EMBL Nucleotide Sequence database (ENA) with accession no. PRJEB44944 (https://www.ebi.ac.uk/ena/browser/view/PRJE44944), which will be available publicly upon acceptance of the article. The RNA-seq data of leaf development were downloaded from the Saccharum Genome database (http://sugarcane.zhangjisenlab.cn/sgd/html/index.html).
