# Supplementary material for: Genomic analysis of SBP gene family in Saccharum spontaneum reveals their association with vegetative and reproductive development
Source: BMC Genomics. 2021 Oct 27;22:767. doi: 10.1186/s12864-021-08090-3 (PMC8549313; doi:10.1186/s12864-021-08090-3)
Supplement: Supplementary file 1 — Additional file 1. [file 12864_2021_8090_MOESM1_ESM.pdf]

Genomic analysis of *SBP* gene family in *Saccharum spontaneum* reveals their association with vegetative and reproductive development

Yanhui Liu [1,2#], Mohammad Aslam[1#], Li-Ang Yao[2], Man Zhang[2], Lulu Wang[2], Huihuang Chen[2], Youmei Huang[2], Yuan Qin[1,2*], Xiaoping Niu[1,2*]

[1]Guangxi Key Laboratory of Sugarcane Biology, State Key Laboratory for Conservation and Utilization of Subtropical Agro-Bioresources, College of Agriculture, Guangxi University, Nanning 530004, China
[2]College of Life Science, Fujian Provincial Key Laboratory of Haixia Applied Plant Systems Biology, Fujian Agriculture and Forestry University, Fuzhou 350002, China

[#] These authors contributed equally to this work.
*Correspondence: yuanqin@fafu.edu.cn; xpniu0613@126.com

Supplemental Figures

Fig. S1. The subcellular localization of SsSBP4 and SsSBP12.

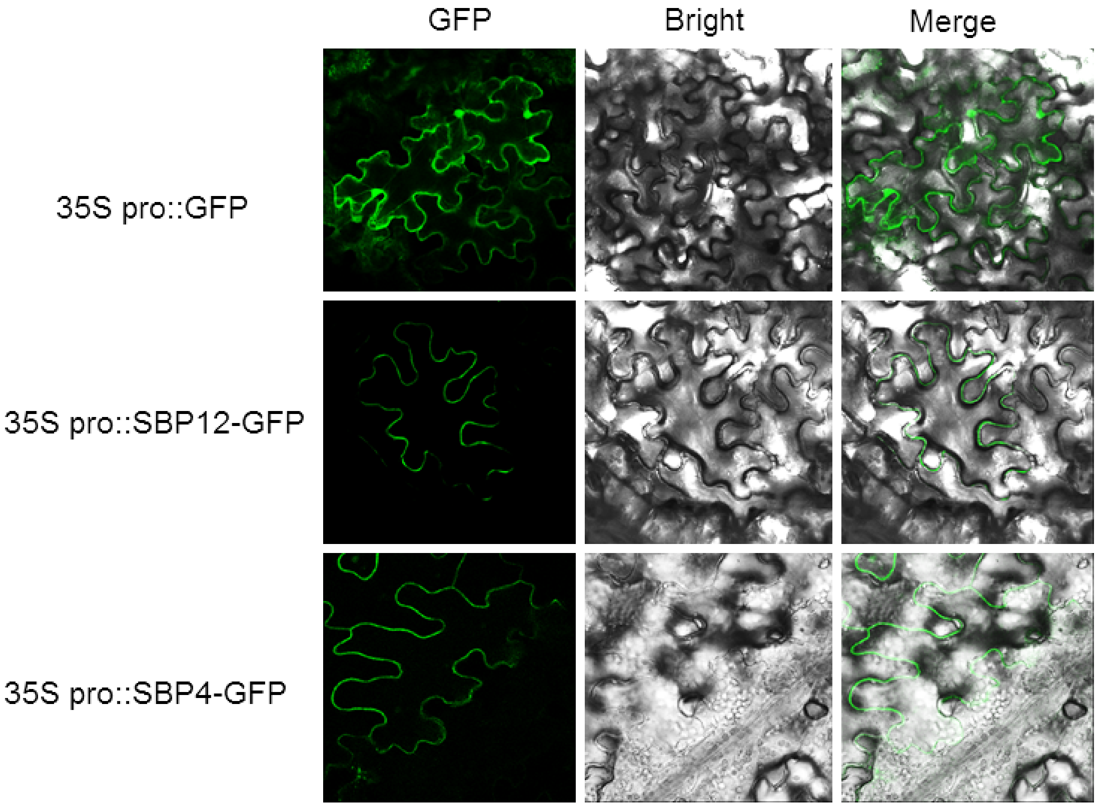

Fig. S2. Sequence logo of 20 conserved motifs of *SBP* gene family in sugarcane.

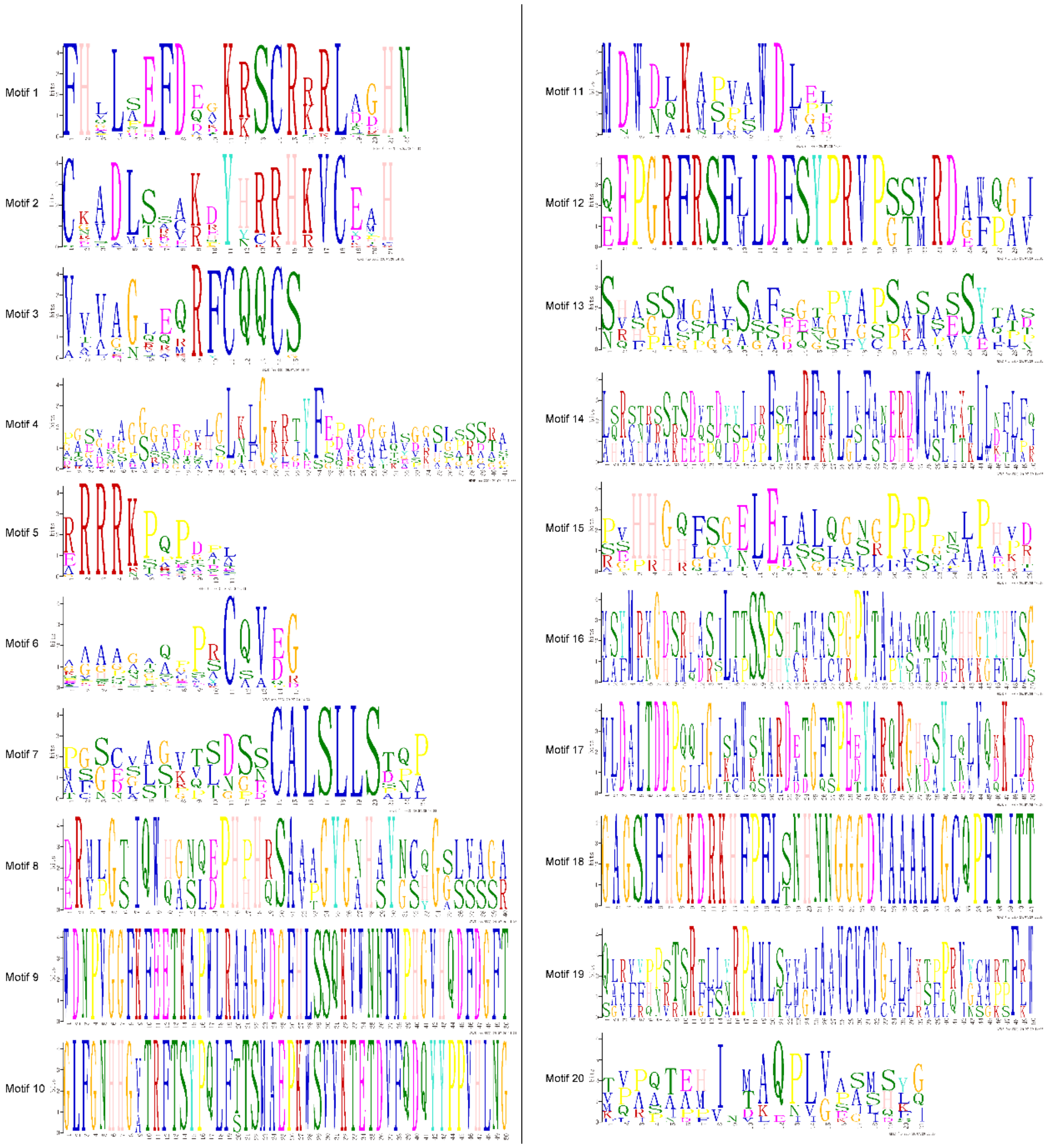

Fig. S3. The distribution of miR156 complementarily binding site.

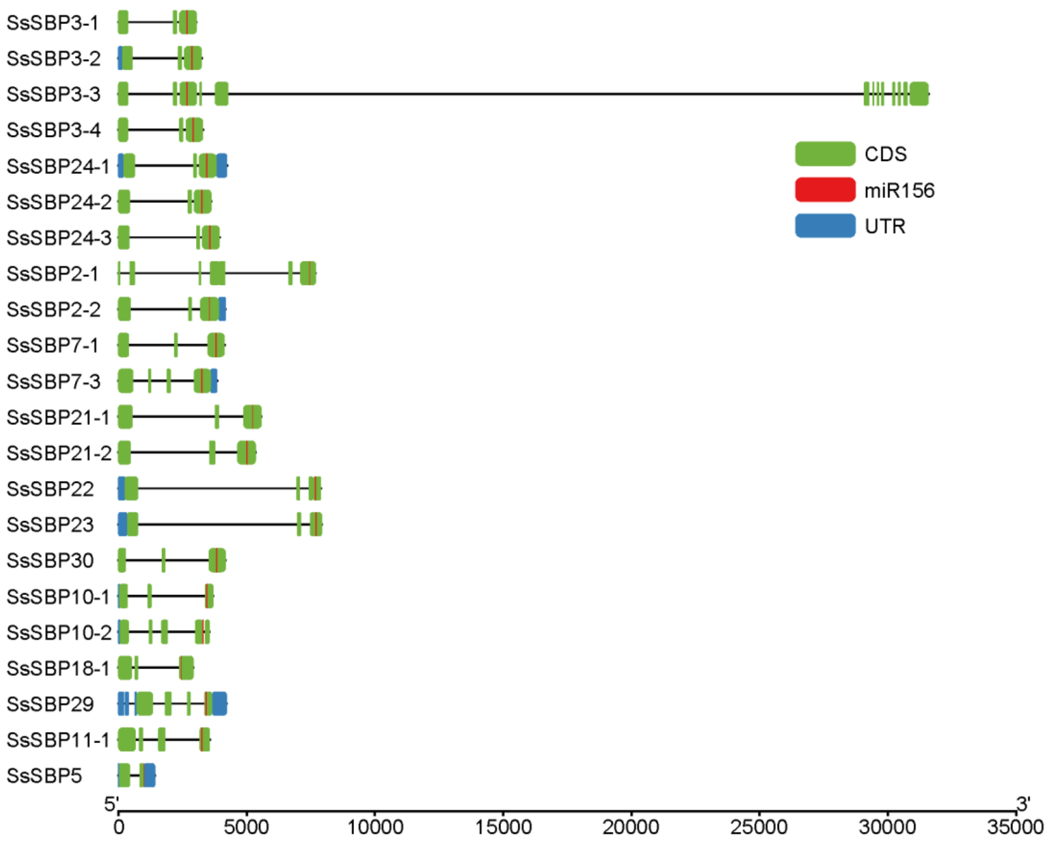

Fig. S4. The distribution of miR156 on the genome of *Saccharum spontaneum*.

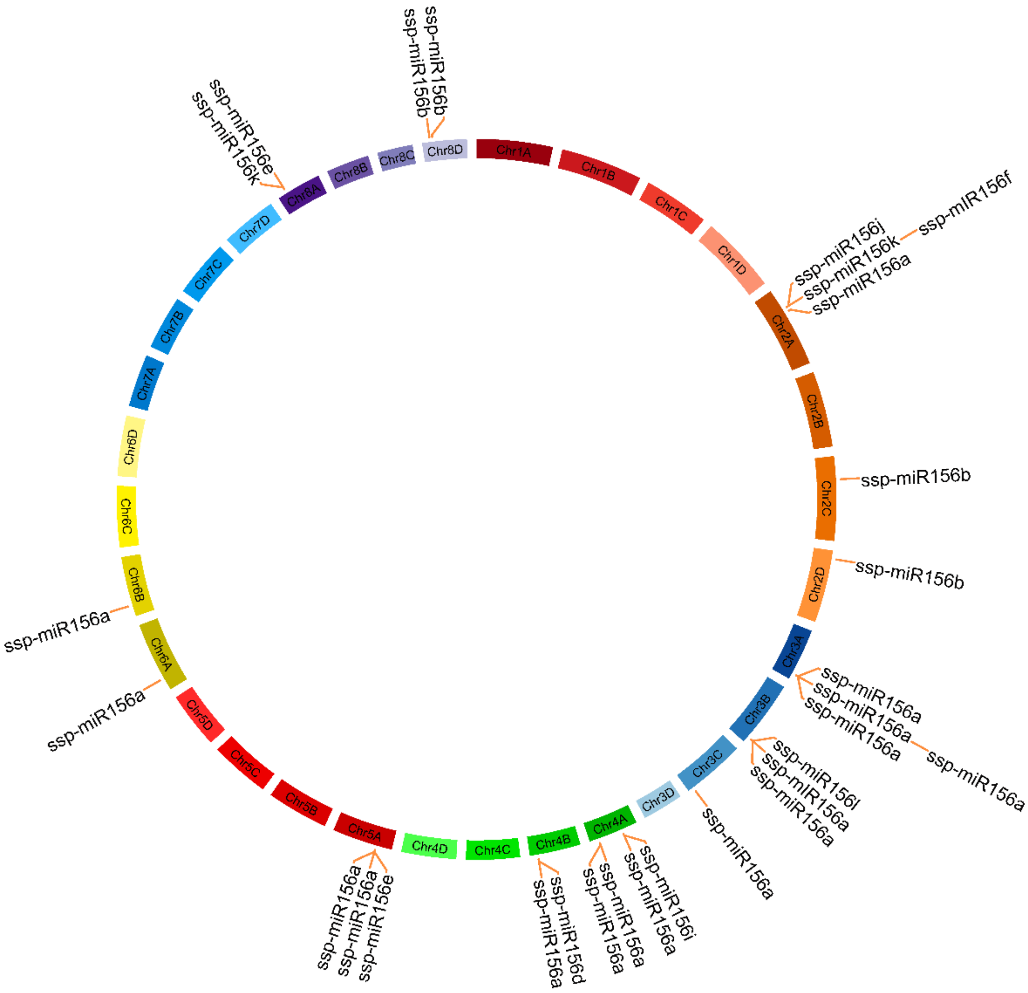

Fig. S5. Quantitative detection of the miR156 precursors expression profiles using RT-PCR and qRT-PCR analysis during the female gametophyte development. The gel images for Fig.S5A-5D were cropped from the original gel image Fig. S5E.

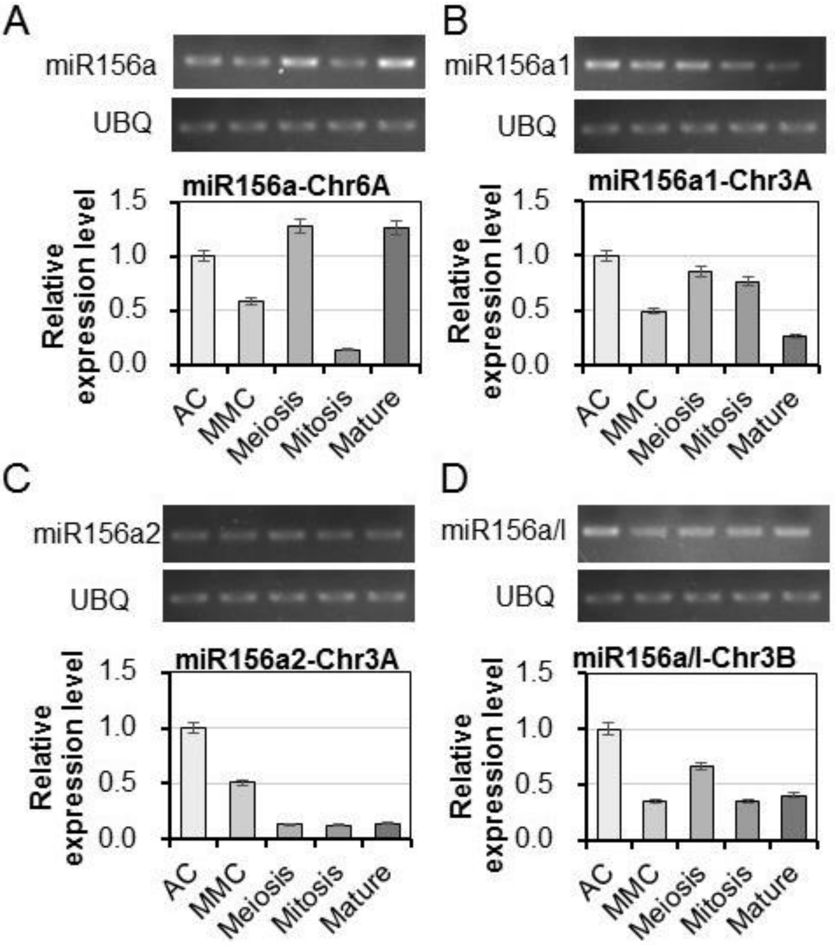

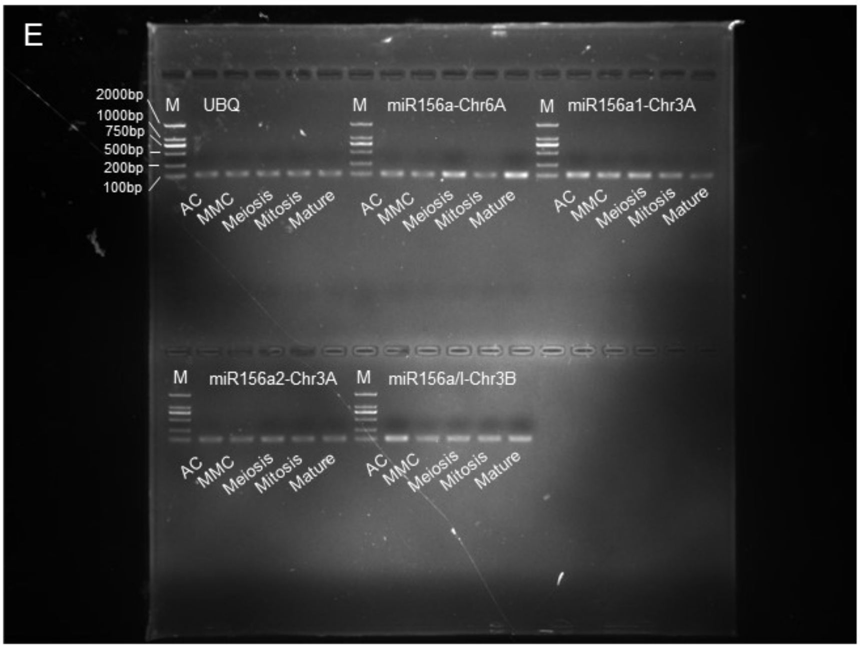

Fig. S6. The expression levels of 4 *SsSBP* genes based on the FPKM values of RNA-seq and qRT-PCR result. Blue line represents for FPKM, and red line represents for qRT-PCR results.

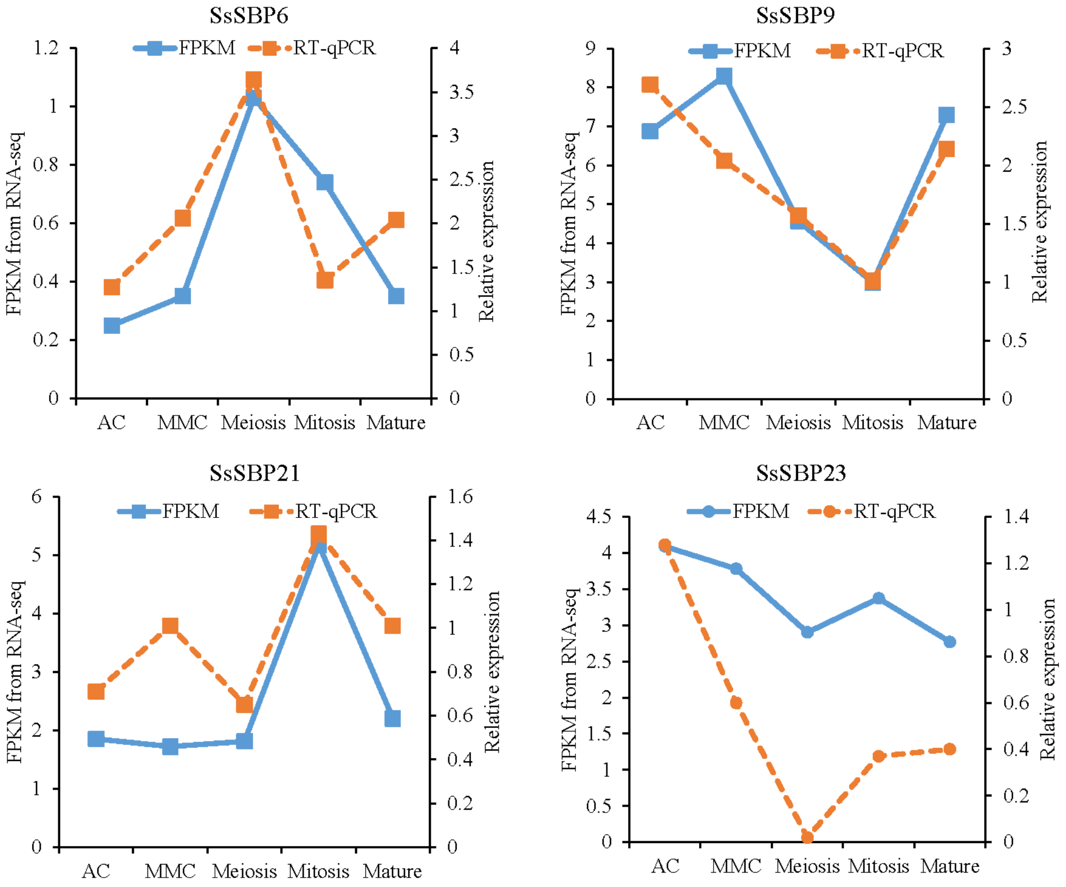

Supplemental Tables

Table S1. Primers used in qRT-PCR analysis.

Gene	Primers (F/R)	Purpose
SsSBP6	ACATGGGCGTGGAGAAG<br>CTGCATTGCTGGCAGAAG	qRT-PCR
SsSBP9	CCTCAGCAGTTGGGAATCAA<br>TATAGGATACGTGGCCTCTCTG	qRT-PCR
SsSBP21	GCACACGATTCTCACCATTTG<br>CAGAGGGATTTGGTGAGTGTAA	qRT-PCR
SsSBP23	TCCGTACCACCAAGGTCTAT<br>CTTGAGAACGATATGGAGAAGGG	qRT-PCR
miR156a-1Chr3A	TGGAGGCTGACAGAAGAGA<br>GAGAGAAGTGAGCACGCATAG	(q)RT-PCR
miR156a/l-Chr3B	CCTCGATCTCTCTTTCGTTCTC<br>CCATGAATAGGTCGTGTCTGT	(q)RT-PCR
miR156a-2Chr3A	TGGAGGCTGACAGAAGAGA<br>GAGAGAAGTGAGCACGCATAG	(q)RT-PCR
miR156a-Chr6A	GTTCCATGGCTAACTGACAGA<br>AGCTGCATCATCATCATTCCTA	(q)RT-PCR

Table S2. Synteny blocks of *SBP* genes within sugarcane.

Gene1	Name1	Gene2	Name2	Ka	Ks	Ka/Ks
Sspon.001A0040480	SsSBP1	Sspon.001D0044800	SsSBP1-2	0.00635569	0.0126072	0.504133
Sspon.002A0013930	SsSBP2-1	Sspon.002B0011930	SsSBP2-2	0.00110024	0.0067091	0.163993
Sspon.002A0015030	SsSBP3-1	Sspon.002B0012730	SsSBP3-2	0.0160542	0.0279504	0.574381
Sspon.002A0015030	SsSBP3-1	Sspon.002C0016120	SsSBP3-3	0.0199181	0.0232536	0.85656
Sspon.002A0013930	SsSBP2-1	Sspon.002D0010972	SsSBP2-3	0.00473868	0.0109806	0.43155
Sspon.002A0015030	SsSBP3-1	Sspon.002D0011720	SsSBP3-4	0.017633	0.0268992	0.65552
Sspon.002A0015030	SsSBP3-1	Sspon.006B0001500	SsSBP24-1	0.248514	0.79158	0.313946
Sspon.002B0012730	SsSBP3-2	Sspon.002C0016120	SsSBP3-3	0.0067972	0.013812	0.492122
Sspon.002B0008220	SsSBP4-1	Sspon.002C0010571	SsSBP5	0.182365	0.175705	1.0379
Sspon.002B0011930	SsSBP2-2	Sspon.002D0010972	SsSBP2-3	0.00325603	0.0033337	0.976694
Sspon.002B0012730	SsSBP3-2	Sspon.002D0011720	SsSBP3-4	0.0137596	0.0069319	1.98497
Sspon.002C0016120	SsSBP3-3	Sspon.002D0011720	SsSBP3-4	0.0137583	0.0139325	0.987492
Sspon.003A0000410	SsSBP7-1	Sspon.003B0003860	SsSBP7-2	0.00113783	0.0067766	0.167908
Sspon.003A0000410	SsSBP7-1	Sspon.003C0005960	SsSBP7-3	0.00113855	0.0135893	0.0837832
Sspon.003B0003860	SsSBP7-2	Sspon.003C0005960	SsSBP7-3	0.0838291	0.108052	0.775821
Sspon.004A0021810	SsSBP10-1	Sspon.004B0022500	SsSBP10-2	0.00346986	0.0053589	0.647497
Sspon.004B0022100	SsSBP13	Sspon.004D0023290	SsSBP15-1	0.107662	0.122687	0.877536
Sspon.006A0002670	SsSBP20-1	Sspon.006C0002070	SsSBP25	0.0648563	0.11707	0.553995
Sspon.006A0003761	SsSBP21-1	Sspon.006D0002410	SsSBP26	0.0959571	0.134648	0.712651
Sspon.006B0001500	SsSBP24-1	Sspon.006C0001780	SsSBP24-2	0.00983246	0.0196958	0.499216
Sspon.006B0001500	SsSBP24-1	Sspon.006D0000820	SsSBP24-3	0.010965	0.0130867	0.837874
Sspon.006C0001780	SsSBP24-2	Sspon.006D0000820	SsSBP24-3	0.00981792	0.0097311	1.00893
Sspon.006C0002070	SsSBP25	Sspon.006D0001140	SsSBP20-2	0.0515641	0.070647	0.729884
Sspon.006D0002410	SsSBP26	Sspon.006D0002450	SsSBP21-2	0.0984319	0.139893	0.703623
Sspon.008B0005752	SsSBP15-2	Sspon.008C0006030	SsSBP15-3	0.00318043	0.0032461	0.97977
Sspon.002D0015430	SsSBP6	Sspon.002D0015440	SsSBP4-3	0.235253	0.304466	0.772675
Sspon.005D0002360	SsSBP17-2	Sspon.005D0002370	SsSBP19	0.0176311	0.0521201	0.338278

Table S3. Synteny blocks of *SBP* genes between sugarcane and sorghum.

Gene1	Name1	Gene2	Name2	Ka	Ks	Ka/Ks
Sspon.001A0040480	SsSBP1-1	Sobic.001G026500	SbSBP1	0.019902804	0.094669056	0.210235576
Sspon.001D0044800	SsSBP1-2	Sobic.001G026500	SbSBP1	0.021251731	0.084831395	0.250517286
Sspon.002A0013930	SsSBP2-1	Sobic.002G257900	SbSBP3	NA	NA	NA
Sspon.002A0013930	SsSBP2-1	Sobic.007G193500	SbSBP13	0.236422811	0.745089664	0.317307866
Sspon.002A0015030	SsSBP3-1	Sobic.002G247800	SbSBP2	0.01683979	0.100982024	0.166760271
Sspon.002B0008220	SsSBP4-1	Sobic.002G312300	SbSBP4_2	NA	NA	NA
Sspon.002B0011930	SsSBP2-2	Sobic.002G257900	SbSBP3	1.185088199	0.708153578	1.673490379
Sspon.002B0011930	SsSBP2-2	Sobic.007G193500	SbSBP13	0.238500431	0.726869921	0.328119825
Sspon.002B0012730	SsSBP3-2	Sobic.002G247800	SbSBP2	0.01933512	0.079630689	0.242809908
Sspon.002C0010571	SsSBP5	Sobic.002G312200	SbSBP4_1	0.201964282	0.285670386	0.706983616
Sspon.002D0010972	SsSBP2-3	Sobic.002G257900	SbSBP3	1.151584789	0.682824118	1.686502803
Sspon.002D0010972	SsSBP2-3	Sobic.007G193500	SbSBP13	0.262012201	0.79212082	0.330773026
Sspon.002D0011720	SsSBP3-4	Sobic.002G247800	SbSBP2	0.014067033	0.074526046	0.18875325
Sspon.003A0000410	SsSBP7-1	Sobic.003G406600	SbSBP6	0.016210589	0.124220009	0.130499014
Sspon.003B0003860	SsSBP7-2	Sobic.003G406600	SbSBP6	0.015040998	0.124220009	0.121083535
Sspon.003C0005960	SsSBP7-3	Sobic.003G406600	SbSBP6	0.013947133	0.120882394	0.115377705
Sspon.003C0027810	SsSBP9	Sobic.003G139900	SbSBP5	NA	NA	NA
Sspon.004A0021810	SsSBP10-1	Sobic.004G058900	SbSBP8	0.006998155	0.079885417	0.087602415
Sspon.004A0023540	SsSBP12	Sobic.004G036900	SbSBP7	0.09450799	0.168573147	0.560634902
Sspon.004B0022500	SsSBP10-2	Sobic.004G058900	SbSBP8	0.009284964	0.095402534	0.097324085
Sspon.004D0023290	SsSBP15-1	Sobic.004G062100	SbSBP9	0.042055111	0.163903531	0.256584533
Sspon.004D0025580	SsSBP11-2	Sobic.004G036900	SbSBP7	1.859997431	2.312592597	0.804291008
Sspon.005A0007540	SsSBP18-1	Sobic.006G171000	SbSBP11	0.044670923	0.12920101	0.345747478
Sspon.005C0003700	SsSBP18-2	Sobic.006G171000	SbSBP11	0.041800602	0.126575085	0.33024352
Sspon.005D0002360	SsSBP17-2	Sobic.006G247700	SbSBP12	0.020330112	0.105252352	0.193155896
Sspon.006A0003761	SsSBP21-1	Sobic.007G193500	SbSBP13	0.014048408	0.106959243	0.131343563
Sspon.006A0019261	SsSBP23	Sobic.005G120600	SbSBP10	NA	NA	NA
Sspon.006B0001500	SsSBP24-1	Sobic.007G210200	SbSBP15	0.995759144	0.951489814	1.046526331
Sspon.006C0001780	SsSBP24-2	Sobic.007G210200	SbSBP15	1.339462291	1.397533825	0.958447135
Sspon.006D0000820	SsSBP24-3	Sobic.007G210200	SbSBP15	1.345200055	1.349566756	0.996764368
Sspon.006D0001140	SsSBP20-2	Sobic.007G207000	SbSBP13	0.043378632	0.180847695	0.239862787
Sspon.006D0002410	SsSBP26	Sobic.007G193500	SbSBP13	0.113175896	0.223912684	0.505446562
Sspon.007A0010700	SsSBP27	Sobic.009G135000	SbSBP16	0.043633064	0.108645104	0.401610958
Sspon.008B0005752	SsSBP15-2	Sobic.004G062100	SbSBP9	0.322490005	0.548898258	0.58752237
Sspon.008B0005752	SsSBP15-2	Sobic.010G215700	SbSBP17	0.025866955	0.068131922	0.379659851
Sspon.008C0006030	SsSBP15-3	Sobic.004G062100	SbSBP9	0.342713236	0.530302784	0.646259546
Sspon.008C0006030	SsSBP15-3	Sobic.010G215700	SbSBP17	0.026497501	0.063565468	0.416853708

Table S4. The distribution information of miR156 obtained in sugarcane.

Name	Chr	miRNA156 sequence	Start	End
Ssp-miR156a	Chr6B	TGACAGAAGAGAGTGAGCAC	17035390	17035506
Ssp-miR156a	Chr4B	TGACAGAAGAGAGTGAGCAC	65310256	65310374
Ssp-miR156d	Chr4B	TGCTCACTTCTCTTTCTGTCAGC	65310256	65310374
Ssp-miR156a1	Chr3A	TGACAGAAGAGAGTGAGCAC	69132148	69132267
Ssp-miR156e	Chr8A	TGACAGAAGAGAGCGAGCAC	13997251	13997373
Ssp-miR156k	Chr8A	GCTCGCTTCTCTTTCTGTCAGC	13997251	13997373
Ssp-miR156a	Chr4A	TGACAGAAGAGAGTGAGCAC	61680304	61680406
Ssp-miR156a	Chr4A	TGTATGTCGTCCTCGCCGTGT	61680304	61680406
Ssp-miR156k	Chr2A	TGACAGAAGAGAGAGAGCAC	32507630	32507753
Ssp-miR156j	Chr2A	GCTCTCTGCTCTCACTGTCATC	32507630	32507753
Ssp-miR156a	Chr5A	TGACAGAAGAGAGTGAGCAC	23781845	23781948
Ssp-miR156a	Chr4A	TGACAGAAGAGAGTGAGCAC	24522774	24522874
Ssp-miR156i	Chr4A	GCTCACTGCTCTATCTGTCATC	24522774	24522874
Ssp-miR156a	Chr2A	TGACAGAAGAGAGTGAGCAC	38830332	38830464
Ssp-mIR156f	Chr2A	TGCTCACTTCTCTTTCTGTCAGC	38830332	38830464
Ssp-miR156a	Chr5A	TGACAGAAGAGAGTGAGCAC	23759443	23759567
Ssp-miR156e	Chr5A	GCTCACTGCTCTCTCTGTCATC	23759443	23759567
Ssp-mIR156a	Chr3B	TGACAGAAGAGAGTGAGCAC	91574690	91575246
Ssp-miR156l	Chr3B	TTGACAGAAGAGAGTGAGCAC	91574690	91575246
Ssp-miR156a2	Chr3A	TGACAGAAGAGAGTGAGCAC	69090925	69091060
Ssp-miR156a	Chr6A	TGACAGAAGAGAGTGAGCAC	19068437	19068569
Ssp-miR156a3	Chr3A	TGACAGAAGAGAGTGAGCAC	69131877	69131960
Ssp-miR156b	Chr2D	TGACAGAAGAGAGTGAGCAC	12099805	12099901
Ssp-miR156a	Chr3C	TGACAGAAGAGAGTGAGCAC	87534474	87534589
Ssp-miR156a4	Chr3A	TGACAGAAGAGAGTGAGCACT	69131914	69132050
Ssp-miR156a	Chr3B	TGACAGAAGAGAGTGAGCACT	91614257	91614409
Ssp-miR156b1	Chr8D	TGACAGAAGAGAGTGAGCACC	11915235	11915378
Ssp-miR156b	Chr2C	TTGACAGAAGATAGAGAGCAC	35047274	35047348
Ssp-miR156b2	Chr8D	ACTCTCTCTCTCTCTGTCTCAAC	16063240	16063384

Note: The miR156a on the chromosome 3A named miR156a1, miR156a2, miR156a3, miR156a4; the miR156b on the chromosome 8D named miR156b1, miR156b2 in Table S7.

Table S5. The expression values of miR156 and its target genes.

Gene	AC	MMC	Meiosis	Mitosis	Mature
SsSBP3-1	0.1837739	0.7253622	0.5755706	1.2403753	0.5199348
SsSBP3-2	1.7776166	1.673006	2.434122	5.8603153	4.2173347
SsSBP3-3	0.4102873	0.2234681	0.5568173	1.3301179	1.0265183
SsSBP3-4	1.1917264	0.8598867	1.4615782	4.4122763	2.8183871
SsSBP24-1	7.917975	5.0556701	6.0172708	10.198655	6.5998454
SsSBP24-2	2.9109158	3.3382741	2.5487349	5.8825076	2.4064033
SsSBP24-3	1.9035845	2.1874165	1.7288847	3.1568787	1.963373
SsSBP2-1	2.919817	2.3247266	1.5426785	4.0073368	3.2424141
SsSBP2-2	1.3730751	2.2354578	2.10018	2.150465	2.1466828
SsSBP7-1	5.7588612	5.3105527	5.7189526	2.940063	1.9139293
SsSBP7-3	24.407098	21.934242	17.792262	11.093469	8.5020534
SsSBP21-1	1.8479265	1.7198743	1.8126177	5.1726938	2.1980383
SsSBP21-2	1.841796	1.1970336	0.9743232	3.6218896	1.084305
SsSBP22	4.7126336	4.5786979	3.5356158	4.5744807	4.5385278
SsSBP23	4.0879269	3.7808772	2.9016029	3.3670189	2.7746782
SsSBP30	18.061099	20.179187	12.990325	7.758196	6.6570913
SsSBP10-1	36.661053	43.934228	40.457009	33.148577	18.758827
SsSBP10-2	3.2177552	2.7790462	2.2653687	3.2926244	1.9360975
SsSBP11-1	7.5281973	8.6455867	4.8237904	3.13388	2.8208172
SsSBP18-1	0	0	0	0	0.0521043
SsSBP29	0.512192	0.1220575	0.3740177	0.5739188	0.4296199
SsSBP5	0.0751788	0.5352439	0.5628228	2.2616017	0.1984631
miR156	128	183	761	334	2539

Note: The reads count represents expression values of miR156.

Table S6. The expression profiles of *SBP* genes across leaf gradients of sugarcane.

Gene	Basai	Transitional	Maturing	Mature
SsSBP1	38.75	33.77	30.32	28.54
SsSBP2	6.84	1.21	0.99	0.47
SsSBP3	1.42	2.76	1.57	0.28
SsSBP4	1.71	0.30	0.50	0.52
SsSBP5	9.58	1.99	3.60	4.85
SsSBP6	1.51	0.27	0.34	0.55
SsSBP7	9.72	3.99	1.55	0.72
SsSBP8	3.60	3.55	2.75	3.25
SsSBP9	3.52	3.27	2.52	3.47
SsSBP10	7.21	5.18	3.56	2.53
SsSBP11	4.70	1.14	0.84	1.17
SsSBP12	7.56	3.42	2.75	3.39
SsSBP13	1.68	0.01	0.00	0.00
SsSBP14	3.13	0.00	0.00	0.00
SsSBP15	4.30	0.00	0.00	0.00
SsSBP16	11.20	6.85	8.01	8.62
SsSBP17	4.49	1.64	0.53	0.47
SsSBP18	0.01	0.00	0.00	0.00
SsSBP19	5.52	2.78	0.75	0.62
SsSBP20	30.23	19.08	14.93	13.68
SsSBP21	2.40	0.33	0.19	0.06
SsSBP22	5.48	4.42	3.55	3.02
SsSBP23	4.72	3.70	2.65	2.58
SsSBP25	22.11	11.22	7.81	9.36
SsSBP24	5.09	0.12	0.11	0.05
SsSBP26	0.35	0.09	0.03	0.01
SsSBP27	6.32	5.91	4.93	5.29
SsSBP28	13.06	12.19	10.51	14.03
SsSBP29	18.99	8.70	6.11	1.72
SsSBP30	30.52	11.63	3.01	0.96

Table S7. The Expression profiles of *SBP* genes in female gametophyte development stages.

Gene	Ac	MMC	Meiosis	Mitosis	Mature
SsSBP1	75.52	83.29	72.53	73.25	70.31
SsSBP4	1.49	1.90	3.05	7.05	2.09
SsSBP2	1.37	2.24	2.10	2.15	2.15
SsSBP5	0.08	0.54	0.56	2.26	0.20
SsSBP3	0.41	0.22	0.56	1.33	1.03
SsSBP6	0.25	0.35	1.03	0.74	0.35
SsSBP8	1.94	2.27	1.35	1.29	3.07
SsSBP7	18.95	16.61	14.94	7.19	7.22
SsSBP9	6.87	8.29	4.56	2.99	7.29
SsSBP10	36.66	43.93	40.46	33.15	18.76
SsSBP12	2.97	2.47	1.65	1.90	1.32
SsSBP13	0.00	0.00	0.00	0.08	0.00
SsSBP14	0.00	0.00	0.00	0.00	0.00
SsSBP15	0.00	0.00	0.00	0.00	0.00
SsSBP11	5.38	6.99	4.19	2.75	2.68
SsSBP16	0.00	0.05	0.00	0.00	0.00
SsSBP17	0.00	0.00	0.00	0.00	0.00
SsSBP18	0.00	0.00	0.00	0.00	0.05
SsSBP19	0.00	0.00	0.00	0.00	0.00
SsSBP20	4.43	7.00	2.12	3.76	5.13
SsSBP21	1.85	1.72	1.81	5.17	2.20
SsSBP22	4.71	4.58	3.54	4.57	4.54
SsSBP23	4.09	3.78	2.90	3.37	2.77
SsSBP25	2.66	3.16	1.17	1.91	3.00
SsSBP24	1.90	2.19	1.73	3.16	1.96
SsSBP26	1.40	1.16	2.26	3.85	2.88
SsSBP27	7.09	3.40	3.88	2.83	2.95
SsSBP28	2.94	4.03	1.79	2.30	2.16
SsSBP29	0.51	0.12	0.37	0.57	0.43
SsSBP30	18.06	20.18	12.99	7.76	6.66
